# A test-time clinically adaptive framework for detecting multiple fundus diseases harnessing ophthalmic foundation models

**DOI:** 10.1038/s41746-026-02480-1

**Published:** 2026-03-02

**Authors:** Hongyang Jiang, Zirong Liu, Mengdi Gao, Bowen Xu, Danqi Fang, Chunwen Zheng, Ruyue Shen, Truong X. Nguyen, An Ran Ran, Clement C. Tham, Qinghua He, Simon K. H. Szeto, Carol Y. Cheung

**Affiliations:** 1 Department of Ophthalmology and Visual Sciences, The Chinese University of Hong Kong, Hong Kong SAR, China; 2 School of Ophthalmology and Optometry and Eye Hospital, Wenzhou Medical University, Wenzhou, China; 3 Department of Ophthalmology, LKS Faculty of Medicine, The University of Hong Kong, Hong Kong SAR, China; 4 College of Chemistry and Life Science, Beijing University of Technology, Beijing, China; 5 Lam Kin Chung. Jet King-Shing Ho Glaucoma Treatment and Research Centre, The Chinese University of Hong Kong, Hong Kong SAR, China; 6 Hong Kong Eye Hospital, Hong Kong SAR, China

**Keywords:** Computational biology and bioinformatics, Diseases, Mathematics and computing, Medical research

## Abstract

Fundus diseases are leading causes of global vision impairment, often presenting with complex comorbidities that challenge conventional artificial intelligence models. While ophthalmic foundation models (FMs) offer promising capabilities, their clinical translation for multi-disease detection remains limited by issues such as imbalanced data distribution, uncertainty in multi-label predictions, inter-disease confusion, and domain shifts. Here, we introduce RetExpert, a test-time clinically adaptive framework that enhances FMs for robust and generalizable detection of multiple fundus diseases from color fundus photographs. RetExpert incorporates adaptive knowledge units with a novel stochastic one-hot activation module to improve generalizability, alongside long-tail-aware learning and an uncertainty-aware multi-label learning strategies. It also integrates a fundus disease co-occurrence matrix as medical prior knowledge to mitigate the confusion score (*C*-score) between diseases. Furthermore, RetExpert employs a lightweight test-time adaptation method combining unsupervised and pseudo-supervised learning (TTUL + TTPL), enabling dynamic parameter adjustment without full retraining. Extensive evaluations on 15 public and private datasets demonstrate that RetExpert outperforms ophthalmic FMs in detection performance, reliability, and cross-domain adaptability, offering a clinically viable solution for automated multi-disease screening in real-world settings.

## Introduction

According to the World Health Organization (WHO) report, approximately 2.2 billion people worldwide suffer from vision impairment or blindness, with fundus diseases such as diabetic retinopathy (DR) (3.9 million), age-related macular degeneration (AMD) (8 million) and glaucoma (7.7 million) accounting for a substantial proportion of these cases^1,2^. Furthermore, fundus diseases in real-world clinical settings often present with comorbid complications^3,4^. This situation highlights the urgent need to develop comprehensive and effective screening approaches capable of early detection, thereby facilitating timely intervention to avoid visual loss. Substantial studies have already reported that artificial intelligence (AI) technologies, including deep learning architectures, convolutional neural networks (CNNs), graph neural networks (GNNs), and vision transformers (ViTs), can be applied to detect fundus diseases from color fundus photographs (CFPs)^5–7^. Nevertheless, conventional methods for simultaneous multi-disease detection in ophthalmology rely heavily on large-scale and finely annotated datasets. Moreover, the integration of multiple specialized models is complex^8^, and single-model solutions continue to experience performance degradation and limited generalizability^9^. These factors restrict the flexibility of such models when transitioning from controlled laboratory environments to diverse clinical settings, ultimately hindering their widespread adoption.

Foundation models (FMs) have emerged as a transformative paradigm in medical AI, demonstrating remarkable potential across various medical imaging applications^10–14^. The paradigm shifts toward data-driven AI was catalyzed by ImageNet^15^, with ImageNet-based pre-trained models serving as early FM prototypes in medical image analysis^16^. Advances in self-supervised learning further enabled the development of general-purpose representations from unlabeled^17^. Recent ophthalmic AI efforts have leveraged large data to develop specialized FMs. In 2023, Zhou et al.^18^ introduced RETFound, the first major ophthalmic FM, trained via masked autoencoder (MAE)^19^ on approximately 1.6 million unlabeled CFPs and optical coherence tomography (OCT) images. RETFound achieves exceptional performance in detecting ocular diseases and predicting systemic diseases, even with limited fine-tuning data. Expending on this work, Qiu et al.^20^ proposed VisionFM, a multimodal FM that incorporates eight ophthalmic imaging modalities. Meanwhile, Julio et al.^21^ developed FLAIR, a vision-language ophthalmic FM based on contrastive language-image pre-training (CLIP)^22^. The model was trained by aligning CFPs with structured textual knowledge from expert annotations, utilizing 37 public datasets, and excelled in few-shot and zero-shot settings.

Despite significant advances in current ophthalmic FMs, their clinical translation remains challenging, particularly for unbiased multi-disease detection within a single model^23^. Specifically, developing a FM-based multi-disease detection model requires deliberate careful consideration to address several key challenges: (1) limited generalizability of the model architecture due to overfitting from insufficient training data in downstream tasks; (2) imbalanced data distribution, multi-disease uncertainty, and inter-disease confusion in multi-disease recognition; (3) domain shift problem due to population out-of-distribution (OOD) in real-world clinical settings. Crucially, strategies to fully exploit the potential of FMs in multi-disease detection remain largely underexplored. Furthermore, the training data for current FMs remains insufficient in scale, leading to compromised generalizability in downstream tasks^24^. This often necessitates repeated fine-tuning across different application scenarios, even for the same task type, to continuously adapt to evolving data domains. Collectively, these challenges significantly increase both model development costs and the barriers to clinical implementation.

**Fig. 1:**
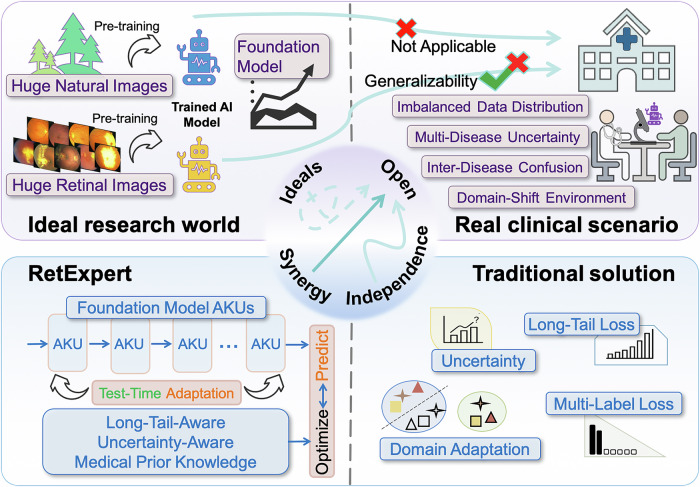
Research overview of clinically translational challenges and solutions in fundus multi-disease AI model. *Top*: Foundation models (FMs) pre-trained on large-scale natural or retinal datasets perform excellent in laboratory settings, their efficacy often diminishes in clinical applications due to comorbidities, uncertainty outputs, and clinical domain shift. *Bottom*: Traditional approaches tackle these challenges in isolation, failing to incorporate an effective synergistic strategy, thereby resulting in a convoluted solution pathway. In contrast, RetExpert leverages the blocks of FMs, constructing them into Adaptive Knowledge Units (AKUs) to enhance generalizability. It further integrates the test-time adaptation (TTA) with long-tail-aware and uncertainty-aware learning strategies, alongside domain-specific prior knowledge to concurrently address key challenges in fundus multi-disease detection: (1) limited generalizability of the model architecture; (2) imbalanced data distribution, multi-disease uncertainty, and inter-disease confusion; (3) domain shift in real-world clinical settings. This yields a domain-adaptive framework tailored for clinical translation. *Centre*: All approaches seek robust open-world performance. The dashed arrow shows an ideal yet unattainable straight route; the pale kinked arrow traces the circuitous traditional path; the solid arrow marks RetExpert’s more direct trajectory.

Therefore, further in-depth research is necessary to elucidate how ophthalmic FM-based approaches can be optimally adapted to enhance fundus multi-disease detection performance. To address clinical translation bottlenecks, we propose RetExpert, a robust and generalizable multiple fundus disease detection model, featuring innovative designs across three key aspects. First, based on the original architecture of ophthalmic FM, we constructed Adaptive Knowledge Units (AKUs) and proposed an adapter-based Stochastic One-hot Activation (SOA) mechanism to enhance both model performance and generalizability. Second, we incorporated long-tail-aware learning with our refined Uncertainty-Aware Multi-label Learning (UAML) strategy, while encoding prior inter-disease relationships into a Fundus Disease Co-occurrence Matrix (FDCM) to address data imbalance, multi-disease uncertainty, and inter-disease confusion. Third, we designed a novel module-wise test-time adaptation (TTA) method to enhance the model’s parameter adaptability under domain shift across diverse clinical scenarios, ultimately improving its feasibility for clinical translation. In summary, a comprehensive visualization of existing research gaps and the key motivations underlying our study is provided in Fig. 1.

## Results

### Performance comparison on internal fundus multi-disease datasets

**Fig. 2:**
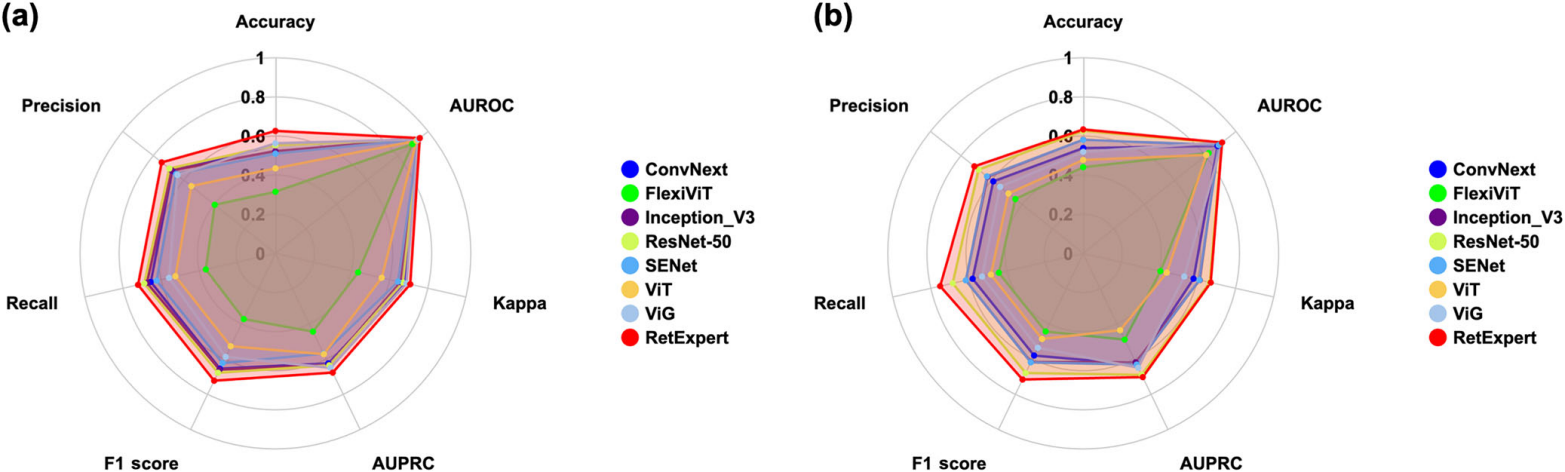
Performance comparison of non-FM-based models against RetExpert. **a** Results on the MuReD dataset. **b** Results on the ODIR dataset.

**Fig. 3:**
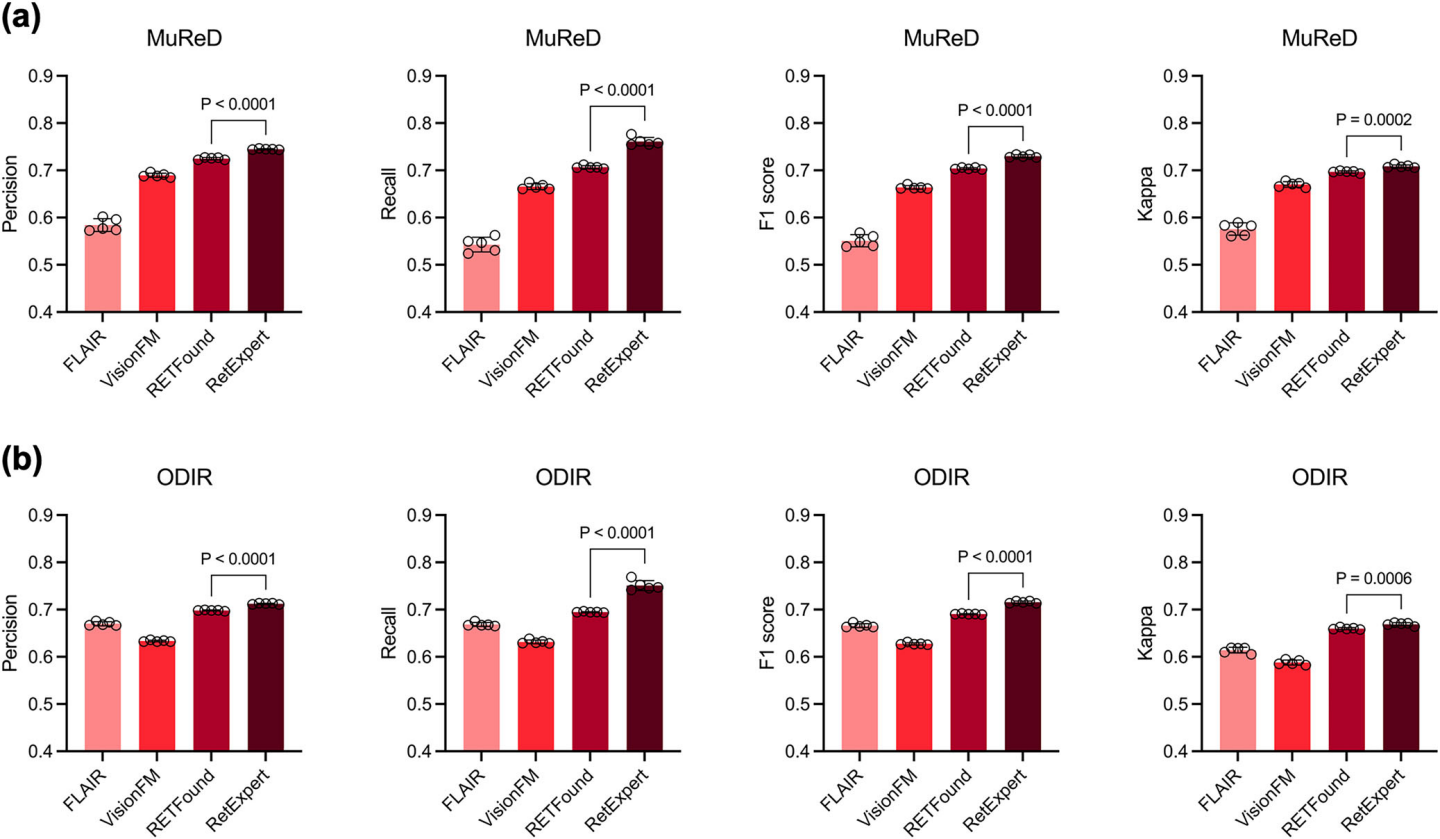
Performance comparison of RetExpert with ophthalmic FMs. **a** Results on the MuReD dataset. **b** Results on the ODIR dataset. The FMs compared include RETFound, FLAIR, and VisionFM.

In this section, the performance of RetExpert was analyzed on two fundus multi-disease datasets, MuReD and ODIR, and compared with state-of-the-art methods. Our evaluation framework encompassed seven distinct performance dimensions, as visualized in Fig. 2. Notably, our analysis incorporated a comprehensive comparison with current advanced model architectures, including ConvNext^25^, FlexViT^26^, Inception V3^27^, ResNet-50^28^, SENet^29^, ViT^30^, and ViG^31^. Each model was initialized with ImageNet pre-trained weights to ensure a fair evaluation. Our experimental results demonstrated that RetExpert consistently outperforms non-FM-based models across all metrics, underscoring the pivotal role of the ophthalmic FM in tackling fundus multi-disease detection challenges (Fig. 2). Furthermore, we compared our RetExpert with recently proposed ophthalmic FMs, including RETFound, FLAIR and VisionFM, on both the MuReD and ODIR datasets, as depicted in Fig. 3. Given the inherent sparsity of disease labels in multi-disease datasets and the realistic clinical fundus multi-disease screening scenario, we prioritized the F1 score and Kappa as the primary evaluation metrics. We further demonstrated that the proposed RetExpert significantly outperforms other ophthalmic FMs, achieving F1 score of 0.7301 (95% CI: 0.7263 to 0.7338) and 0.7156 (95% CI: 0.7126 to 0.7186), and Kappa value of 0.70844 (95% CI: 0.7045 to 0.7123) and 0.6691 (95% CI: 0.6652 to 0.6730) on the MuReD and ODIR datasets, respectively.

### Effectiveness of fine-tuning approach and training strategy for RetExpert

We evaluated the effectiveness of the embedded adapters within the AKU and assessed the impact of key modules within RetExpert, including SOA and UAML.

**Fig. 4:**
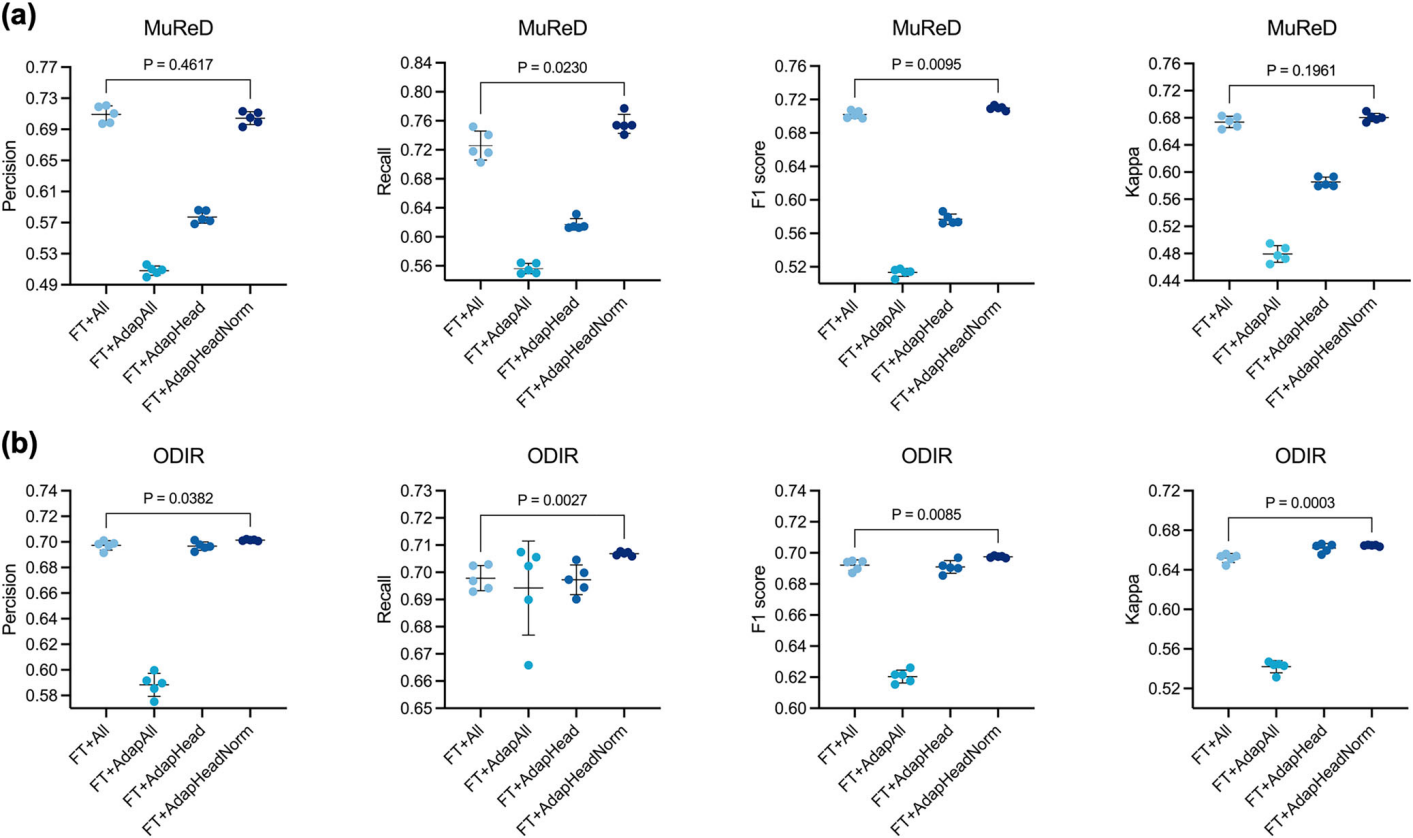
Comparison of fine-tuning approaches for AKUs in RetExpert. **a** Results on the MuReD dataset. **b** Results on the ODIR dataset.

Fine-tuning approach analysis. Four fine-tuning strategies were conducted for comparison: FT+All refers to the conventional full fine-tuning approach; FT + AdapAll involves fine-tuning all parameters after integrating adapters; FT + AdapHead denotes fine-tuning only the adapters within AKU and the task-specific head; and FT + AdapHeadNorm extends this by also including the normalization layers. Comparative results were shown in Fig. 4, which indicated that FT + AdapHeadNorm achieved significantly superior F1 scores of 0.7097 (95% CI: 0.7062 to 0.7132, *P* < 0.01) and 0.6975 (95% CI: 0.6966 to 0.6983, *P* < 0.01) on both datasets, while maintaining Kappa values comparable to those of the FT + All. Other adapter-based fine-tuning approaches (i.e., FT + AdapAll and FT + AdapHead) did not produce any performance improvements. These findings highlighted that the adapter-based fine-tuning achieved better and more stable performance, while underscoring the importance of selecting an appropriate fine-tuning approach to enhance the performance of ophthalmic FMs in fundus multi-disease detection.

**Fig. 5:**
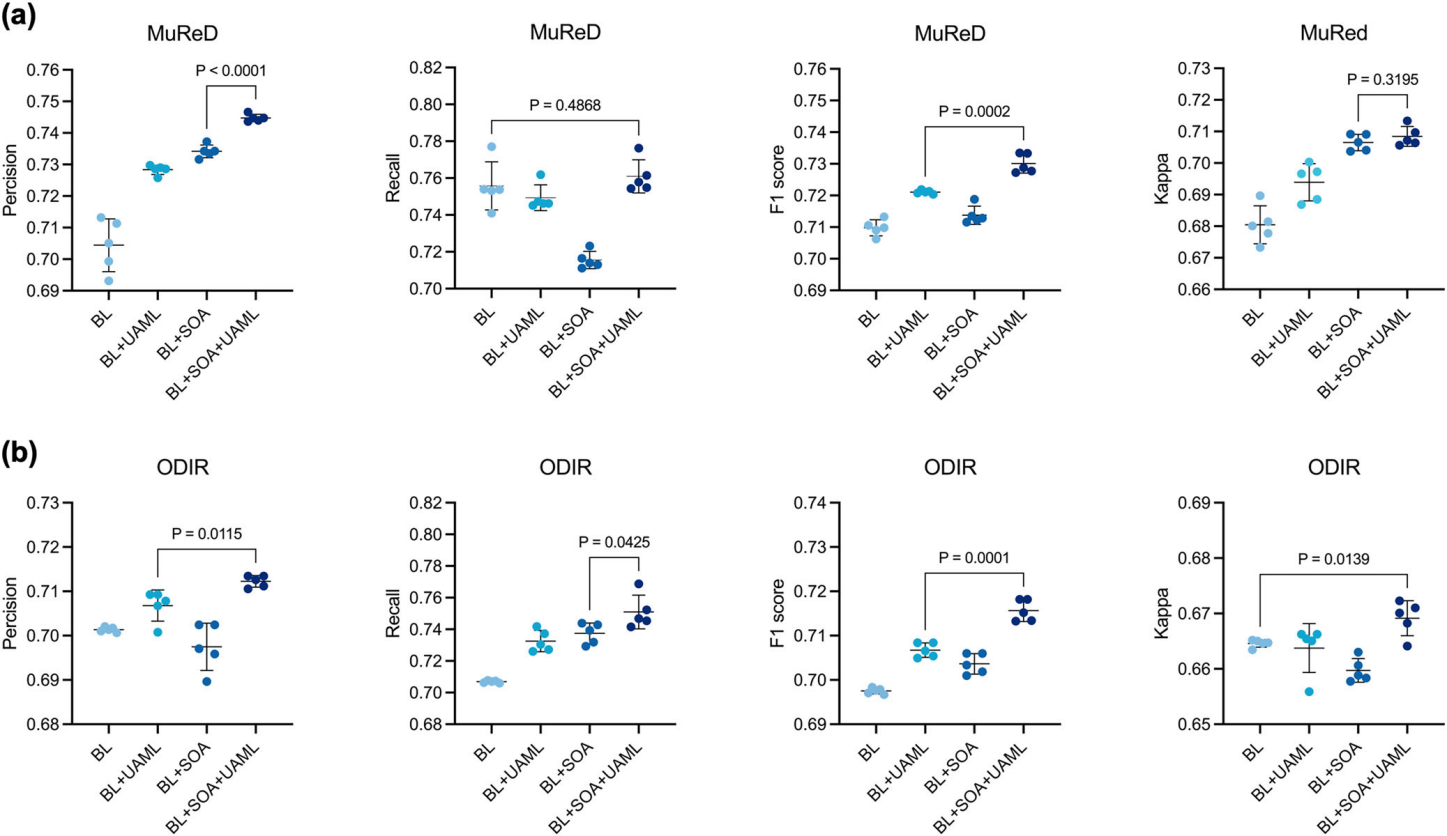
Ablation analysis of the SOA and UAML modules in RetExpert. **a** Results on the MuReD dataset. **b** Results on the ODIR dataset. BL (baseline) denotes the adapter-based fine-tuning method (FT + AdapHeadNorm).

**Fig. 6:**
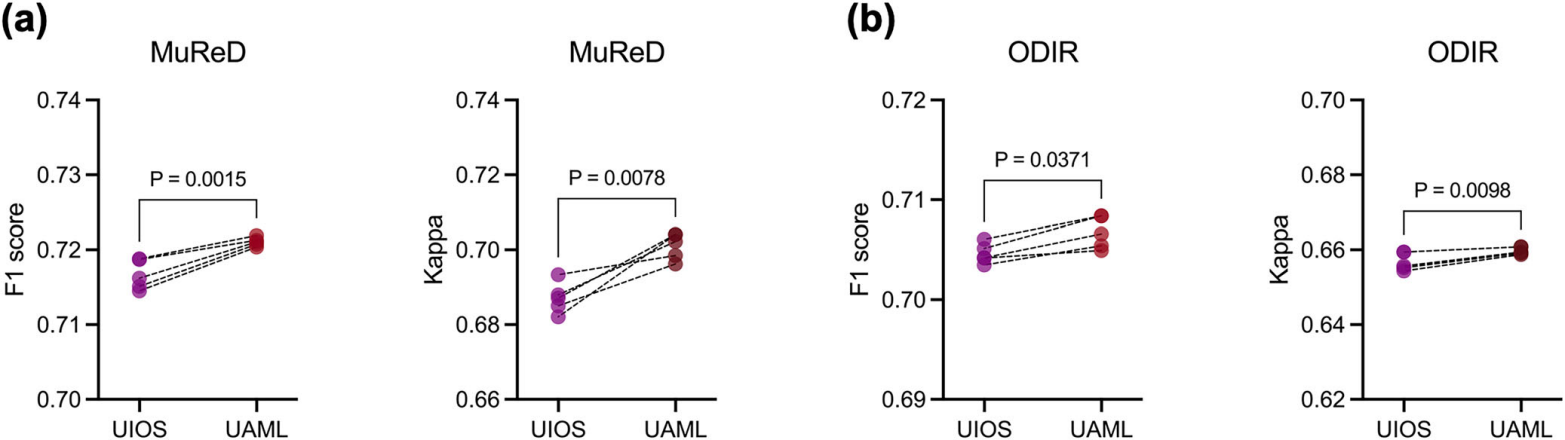
Comparison of F1 score and Kappa between the UIOS and UAML methods. Both UIOS and UAML were evaluated under baseline experimental settings. **a** Results on the MuReD dataset. **b** Results on the ODIR dataset.

Key module analysis. We conducted ablation studies on our proposed crucial SOA and UAML modules within RetExpert across the two datasets, as demonstrated Fig. 5. The baseline (BL) is the adapter-based fine-tuning approach (FT + AdapHeadNorm) presented in Fig. 4. Experimental results demonstrated that, compared with BL, individual application of either the SOA or UAML module further enhances RetExpert’s performance on the MuReD and ODIR datasets. Specifically, using SOA alone achieved F1 scores of 0.7137 (95% CI: 0.7101 to 0.7173, *P* = 0.04) and 0.7036 (95% CI: 0.7007 to 0.7065, *P* < 0.01) on the MuReD and ODIR datasets, respectively, significantly outperforming those of the BL. Similarly, UAML alone yielded F1 scores of 0.7210 (95% CI: 0.7203 to 0.7217, *P* < 0.01) and 0.7067 (95% CI: 0.7047 to 0.7087, *P* < 0.01) on the same datasets, which also significantly exceeded the BL. When both SOA and UAML were applied simultaneously, RetExpert exhibited a further improvement among all metrics, with a particularly significant increase in F1 score: 0.7301 (95% CI: 0.7263 to 0.7338, *P* < 0.01) and 0.7156 (95% CI: 0.7126 to 0.7186, *P* < 0.01) on two datasets, respectively. These results highlighted the potential to further enhance model performance by optimizing training strategies from the perspectives of model generalizability and multi-disease uncertainty. Furthermore, we conducted a comparative experiment on uncertainty (Fig. 6), contrasting the naive single-value UIOS with our modified multi-value UAML. Under the baseline experimental settings, the proposed UAML acquired F1 scores of 0.7210 (95% CI: 0.7203 to 0.7218, *P* < 0.01) and 0.7067 (95% CI: 0.7049 to 0.7085, *P* < 0.01), and Kappa values of 0.7010 (95% CI: 0.6972 to 0.7048, *P* < 0.05) and 0.6598 (95% CI: 0.6586 to 0.6610, *P* < 0.01), on two datasets respectively, was significantly better than those of UIOS. Moreover, by leveraging its multi-disease uncertainty outputs, it would serve as an effective filter for disease categories in our TTA algorithm (Supplementary Note 2), thereby enhancing model robustness on unseen clinical datasets, as presented in Section “Test-time adaptive validation across domain shifts”.

### Inter-disease confusion analysis of RetExpert

**Fig. 7:**
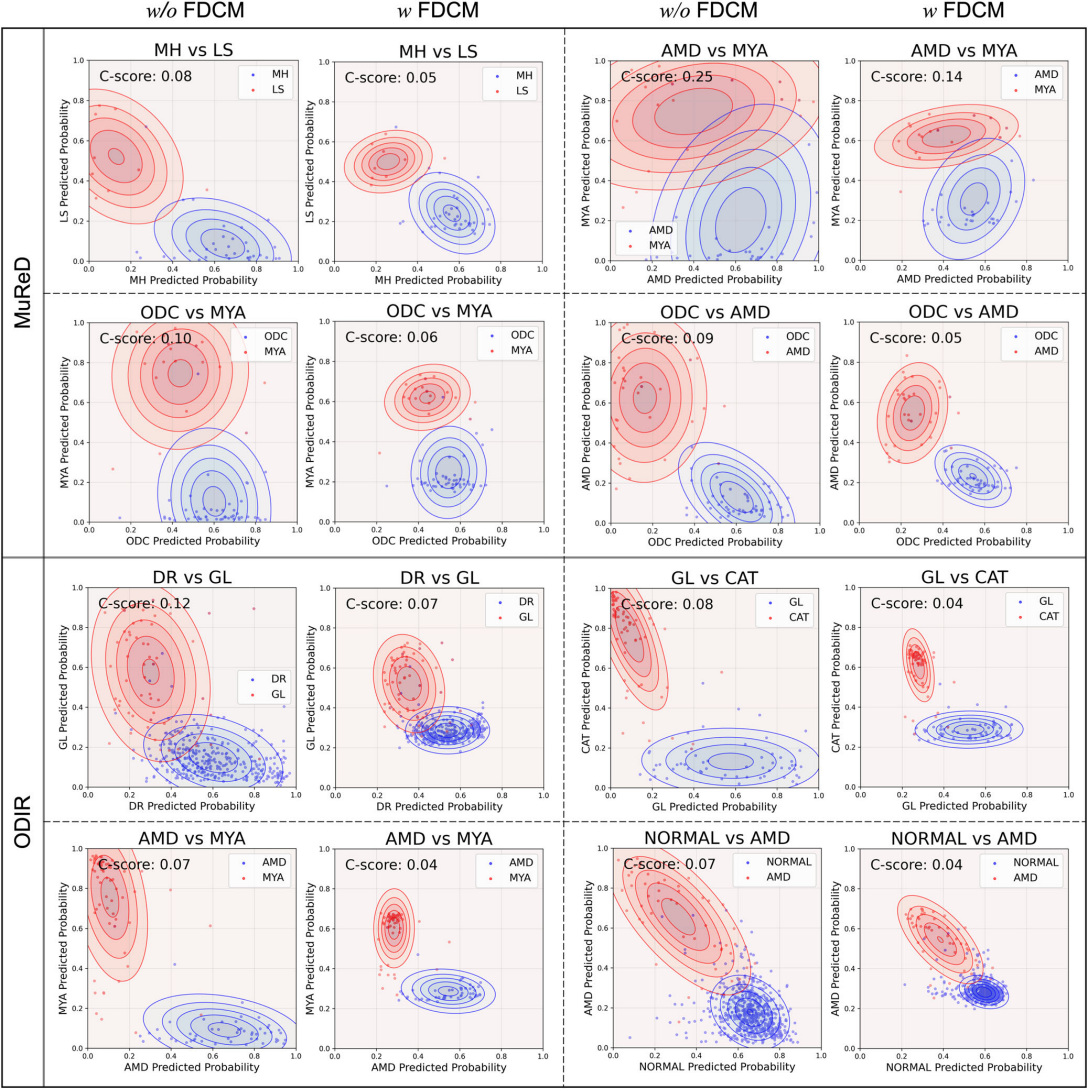
Comparison of probability scatter plot examples with *C*-score predicted by RetExpert. Both conditions, with (*w*) and without (*w/o*) FDCM, were evaluated across the MuReD and ODIR datasets. A lower *C*-score for a given disease pair indicates higher predictive reliability of the model's output, whereas a higher *C*-score suggests lower reliability. All probability scatter plots of the *C*-score are provided in Supplementary Figs. 3–10.

In conventional fundus multi-disease classification, previous research has primarily emphasized standard performance metrics (e.g., F1 score and Kappa), yet seldom addressed inter-disease confusion. Analyzing the *C*-score is essential for reliably evaluating models, especially those destined for real-world clinical use. Accordingly, in this study, we conducted a comparative analysis of the *C*-score for RetExpert. For example, in fundus multi-disease detection, it is clinically inappropriate for a multi-disease detection model to produce conflicting predictions, such as simultaneously classifying a case as both “Normal” and “AMD”. The proposed FDCM regularizes the relationships between fundus diseases to some extent, thereby reducing the degree of inter-disease confusion in RetExpert. As illustrated in Fig. 7, we present multiple paired visualization examples to demonstrate the *C*-score of RetExpert after integrating the FDCM on both the MuReD and ODIR datasets. The sample prediction probabilities of RetExpert with FDCM exhibit a distinct centralization trend, characterized by smaller variance and a more stable probability prediction range. Importantly, the FDCM enabled RetExpert to achieve a lower *C*-score, with a reduction of over 35% across multiple disease pairs. Specifically, on the MuReD dataset, RetExpert (*w* FDCM) reduced the *C*-score between AMD and myopia (MYL) from 0.25 to 0.14 (a 44% decrease); on the ODIR dataset, RetExpert (*w* FDCM) reduced the *C*-score between glaucoma (GL) and cataract (CAT) from 0.08 to 0.04 (a 50% decrease). Experimental results indicated that the FDCM effectively mitigates the confusion in RetExpert across all pairs of fundus diseases without compromising the model’s overall performance. Comprehensive comparisons of all visualized probability scatter plots are provided in Supplementary Figs. 3–10.

### Test-time adaptive validation across domain shifts

**Fig. 8:**
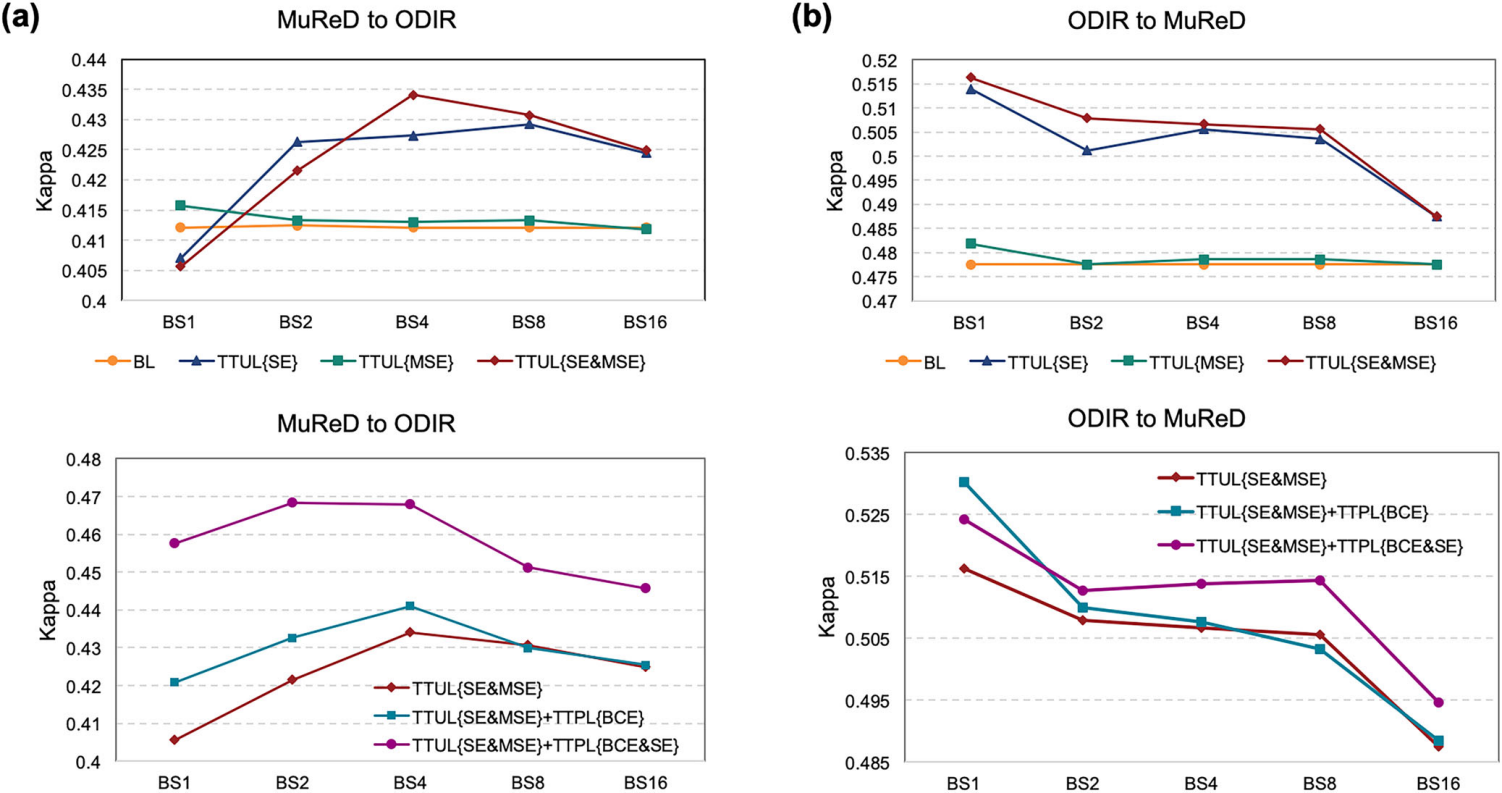
Ablation study on TTUL and TTPL stages during cross-domain TTA. **a** MuReD to ODIR domain shift. **b** ODIR to MuReD domain shift. Performance is compared across different loss functions (MSE, SE, BCE) and batch sizes(BS).

**Fig. 9:**
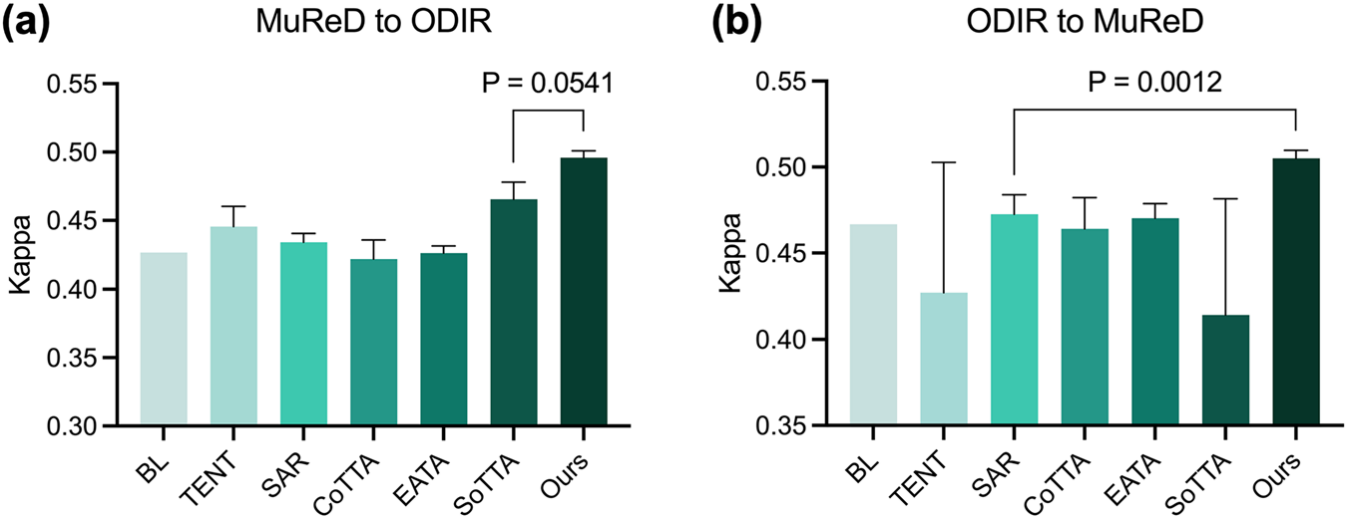
Performance comparison of state-of-the-art TTA methods within the RetExpert framework. **a** MuReD to ODIR domain shift. **b** ODIR to MuReD domain shift.

We systematically evaluated the effectiveness of the proposed TTUL and TTPL for test-time domain adaptation via ablation studies, considering bidirectional domain shifts between MuReD and ODIR under varying batch sizes (1, 2, 4, 8, and 16). In our study, since a single iteration over *K* and *I* (Algorithm in Supplementary Note 2) was sufficient for performance improvement, all results for TTUL and TTPL are therefore reported with *K* = 1 and *I* = 1. For fundus multi-disease detection, Kappa was adopted as the primary evaluation metric to assess different approaches, with results presented in both Figs. 8 and 9. In the TTUL stage, we investigated the effects of MSE loss and SE loss on the AKU’s domain adaptation. The results showed that combining MSE and SE losses achieved the highest mean kappa values of 0.4233 (95% CI: 0.4096 to 0.4370) and 0.5047 (95% CI: 0.4916 to 0.5178) across varying batch sizes on both domain-shift datasets. Optimal performance was observed at batch sizes of four and one, respectively. In the TTPL stage, we further fine-tuned the task-specific head based on TTUL through pseudo-supervised learning for domain adaptation. The results in Fig. 8, indicate that incorporating a multi-label uncertainty score generated by UAML as a weighting coefficient into binary cross-entropy (BCE) loss significantly improves the overall performance of the task-specific head across two domain-shift scenarios. Additionally, combining BCE loss with SE loss further enhanced the model performance significantly, achieving peak mean Kappa values of 0.4582 (95% CI: 0.4457 to 0.4706) and 0.5119 (95% CI: 0.4986 to 0.5252) across batch sizes for the two datasets. Notably, the model attained its best performance at batch sizes of two and one, respectively. A batch size of one is particularly significant as it simulates a realistic clinical diagnostic setting where only one fundus image is available at a time. Details regarding inference time and computational resource consumption are provided in Supplementary Table 1. In this challenging scenario, we confirmed that RetExpert maintains strong performance, especially in the ODIR to MuReD adaptation.

To further validate the superiority of our TTA framework, we conducted comparative experiments against state-of-the-art methods, including TENT^32^, SAR^33^, CoTTA^34^, EATA^35^, and SoTTA^36^. As illustrated in Fig. 9, our method demonstrated superior performance gains in both domain adaptation tasks (MuReD to ODIR, *P* = 0.0541; ODIR to MuReD, *P* = 0.0012), outperforming all benchmark methods.

### Performance evaluation in unseen clinical datasets

To better approximate real-world clinical screening scenarios, nine publicly available CFP datasets representing diverse imaging domains were curated, comprising both single-disease and multi-disease cases for comprehensive validation. Comparative experiments across multiple public datasets are presented in Table 1. Specifically, the ADAM, iChallenge-GON, PALM, GAMMA, and MMAC datasets, all originating from China, were treated as population OOD datasets. In contrast, the HRF and OTFID datasets, which were acquired using devices substantially different from those in MuReD, were considered device OOD datasets. For the AMD detection task (ADAM), RetExpert achieved a higher AUROC of 0.9600 (95% CI: 0.9360 to 0.9770) and an AUPRC of 0.8905 (95% CI: 0.8065 to 0.9405), surpassing all comparison methods across additional metrics. Similarly, in the referable DR detection task (DRarranged), RetExpert comprehensively obtained significantly superior performance, with an AUROC of 0.8983 (95% CI: 0.8951 to 0.9012) and an AUPRC of 0.7910 (95% CI: 0.7810 to 0.8010). For glaucoma detection tasks, RetExpert also demonstrated robust results, delivering the highest reported AUROC values of 0.8689 (95% CI: 0.7864 to 0.9274), 0.9466 (95% CI: 0.8828 to 0.9818), and 0.9122 (95% CI: 0.8806 to 0.9386) on the Drishti-GS, GAMMA, and GON400 datasets, respectively. Although RetExpert’s improvement in AUROC over RETFound did not reach statistical significance, it demonstrated consistent superiority across other key metrics, including F1 score, Kappa, *C*-score, and AUPRC. Notably, RetExpert’s lower *C*-score reflects its reduced susceptibility to catastrophic confusion, a critical feature for clinical reliability. For the task of detecting myopic retinal changes, we evaluated RetExpert on two datasets (i.e., MMAC and PALM), where it consistently achieved leading performance, with AUROC values of 0.9713 (95% CI: 0.9602 to 0.9804) and 0.9853 (95% CI: 0.9682 to 0.9854), and AUPRC values of 0.9852 (95% CI: 0.9742 to 0.9922) and 0.9908 (95% CI: 0.9658 to 0.9978), respectively. These results demonstrate that RetExpert further pushes performance boundaries even for relatively straightforward retinal disease detection tasks, underscoring its generalizability across varying clinical complexities. Furthermore, we evaluated RetExpert on ocular toxoplasmosis (OT) cases, which should be categorized as “other” in our learning tasks. Experimental results showed that RetExpert achieved outstanding OT detection performance, attaining an AUROC of 0.9842 (95% CI: 0.9672 to 0.9942) and an AUPRC of 0.9931 (95% CI: 0.9721 to 0.9981), with statistically significant superiority over all comparative models. We further evaluated RetExpert on a small sample-size multi-disease dataset (i.e., HRF, *n* = 45) comprising normal, DR, and glaucoma cases, achieving a mean AUROC of 0.7973 and a mean AUPRC of 0.7377, which was marginally superior to VisionFM. Notably, RetExpert outperformed VisionFM by over 10 percentage points in Kappa, a critical metric for multiclass evaluation, demonstrating its robust performance in real-world multiclass disease detection scenarios.

**Table 1:** Unseen open-access clinical datasets for validating baseline FMs and our RetExpert model

Test on ADAM	F1 score	Kappa	*C*-score	AUPRC	AUROC	*P*-value
FLAIR	0.0807	0.0678	0.3698	0.3475 ± 0.0453	0.6314 ± 0.0338	<0.001
VisionFM	0.7651	0.4043	0.1821	0.7289 ± 0.0424	0.8570 ± 0.0223	<0.001
RETFound	0.7977	0.4354	0.2023	0.7829 ± 0.0364	0.8780 ± 0.0232	<0.001
RetExpert	**0.8824**	**0.6333**	**0.0580**	**0.8905** ± **0.0252**	**0.9600** ± **0.0085**	Ref.
**Test on DRarranged**	F1 score	Kappa	*C*-score	AUPRC	AUROC	*P*-value
FLAIR	0.6667	0.1137	0.4404	0.3456 ± 0.0059	0.6314 ± 0.0038	<0.001
VisionFM	0.7415	0.2462	0.5727	0.4653 ± 0.0061	0.7016 ± 0.0037	<0.001
RETFound	0.7857	0.3275	0.4808	0.6087 ± 0.0055	0.7729 ± 0.0035	<0.001
etExpert	**0.8633**	**0.3362**	**0.0717**	**0.7910** ± **0.0044**	**0.8983** ± **0.0027**	Ref.
Test on Drishti-GS	F1 score	Kappa	*C*-score	AUPRC	AUROC	*P*-value
FLAIR	0.6931	0.1329	0.6421	0.7696 ± 0.0579	0.6287 ± 0.0601	0.001
VisionFM	**0.7930**	0.4609	0.3212	0.8751 ± 0.0469	0.8087 ± 0.0539	0.047
RETFound	0.6842	0.3532	0.3502	0.8990 ± 0.0039	0.8479 ± 0.0475	0.372
RetExpert	0.7803	**0.5046**	**0.1474**	**0.9316** ± **0.0247**	**0.8689** ± **0.0388**	Ref.
Test on GAMMA	F1 score	Kappa	*C*-score	AUPRC	AUROC	*P*-value
FLAIR	0.6667	0.1700	0.5298	0.6990 ± 0.0698	0.6951 ± 0.0524	<0.001
VisionFM	0.7172	0.4400	0.3142	0.7743 ± 0.0716	0.8540 ± 0.0389	0.022
RETFound	0.7773	0.5300	0.1025	0.9333 ± 0.0286	0.9241 ± 0.0283	0.117
RetExpert	**0.8792**	**0.7500**	**0.1917**	**0.9537** ± **0.0204**	**0.9466** ± **0.0228**	Ref.
Test on iChallengeGON	F1 score	Kappa	*C*-score	AUPRC	AUROC	*P*-value
FLAIR	0.6667	0.1150	0.6025	0.2265 ± 0.0561	0.6525 ± 0.0480	<0.001
VisionFM	0.8622	0.2186	0.3733	0.2459 ± 0.0582	0.6972 ± 0.0448	<0.001
RETFound	0.8965	0.4156	0.3077	0.6969 ± 0.0669	0.8835 ± 0.0349	0.104
RetExpert	**0.9001**	**0.4585**	**0.2593**	**0.7105** ± **0.0643**	**0.9122** ± **0.0257**	Ref.
Test on MMAC	F1 score	Kappa	*C*-score	AUPRC	AUROC	*P*-value
FLAIR	0.6667	0.1054	0.4775	0.7415 ± 0.0320	0.5711 ± 0.0343	<0.001
VisionFM	0.7774	0.5529	0.0837	0.9742 ± 0.0065	0.9452 ± 0.0118	<0.001
RETFound	0.8245	0.6235	0.0872	0.9798 ± 0.0053	0.9575 ± 0.0108	<0.001
RetExpert	**0.8537**	**0.6587**	**0.0581**	**0.9852** ± **0.0046**	**0.9713** ± **0.0083**	Ref.
Test on PALM	F1 score	Kappa	*C*-score	AUPRC	AUROC	*P*-value
FLAIR	0.6667	0.1818	0.1109	0.7970 ± 0.0272	0.7395 ± 0.0245	<0.001
VisionFM	0.8796	0.7642	0.0747	0.9728 ± 0.0055	0.9498 ± 0.0102	<0.001
RETFound	0.8869	0.7762	0.0738	0.9871 ± 0.0039	0.9746 ± 0.0076	0.101
RetExpert	**0.9495**	**0.8995**	**0.0250**	**0.9908** ± **0.0026**	**0.9853** ± **0.0042**	Ref.
**Test on OTFID**	F1 score	Kappa	*C*-score	AUPRC	AUROC	*P*-value
FLAIR	0.6990	0.1846	0.1816	0.7869 ± 0.0244	0.6138 ± 0.0289	<0.001
VisionFM	0.8094	0.5756	0.1828	0.9212 ± 0.0169	0.8692 ± 0.0189	<0.001
RETFound	0.9093	0.8037	0.0606	0.9886 ± 0.0034	0.9744 ± 0.0073	0.025
RetExpert	**0.9382**	**0.8599**	**0.0593**	**0.9931** ± **0.0023**	**0.9842** ± **0.0053**	Ref.
**Test on HRF**	F1 score	Kappa	*C*-score	AUPRC	AUROC	*P*-value
FLAIR	0.5000	0.2648	0.4025	0.5952 ± 0.0952	0.7150 ± 0.0784	0.053
VisionFM	0.6154	0.3944	0.3319	0.7014 ± 0.1016	0.7754 ± 0.0805	0.129
RETFound	0.5714	0.3365	0.5835	0.6185 ± 0.1088	0.6451 ± 0.0968	0.066
RetExpert	**0.6591**	**0.4984**	**0.3204**	**0.7377** ± **0.0962**	**0.7973** ± **0.0691**	Ref.

A detailed description of these datasets is provided in Supplementary Table 2.

Bold values indicate the best performance.

Finally, we assembled retrospective multicenter datasets from various clinical settings across Hong Kong, Mainland China, and Vietnam to validate the generalizability of RetExpert, as presented in Table 2. All these datasets were population OOD datasets, and the Hand-Held dataset, acquired with a portable camera, was considered a representative device OOD dataset. Across three datasets for the referable DR detection task, namely STDR, Vietnam, and AITS, our method achieved AUROC values of 0.8572 (95% CI: 0.8512 to 0.8642), 0.8477 (95% CI: 0.8346 to 0.8618), and 0.8417 (95% CI: 0.8188 to 0.8636), respectively, along with AUPRC values of 0.8936 (95% CI: 0.8854 to 0.9018), 0.6278 (95% CI: 0.5720 to 0.6738), and 0.5590 (95% CI: 0.4730 to 0.6460). Although RetExpert demonstrated superior DR detection performance compared to other ophthalmic FMs, it still exhibited a performance gap relative to specialized DR detection systems, highlighting the inherent challenges in integrating multi-disease detection capabilities within a single unified model. Furthermore, to validate the robustness of RetExpert, we evaluated its performance on two CFP datasets with distinct FOVs for glaucoma detection: CUHK-GON(D) (disc-centered) and CUHK-GON(M) (macula-centered). RetExpert demonstrated stronger and more consistent performance across both datasets, achieving AUROC values of 0.9547 (95% CI: 0.9492 to 0.9602) and 0.9594 (95% CI: 0.9506 to 0.9682), with corresponding AUPRC values of 0.9916 (95% CI: 0.9905 to 0.9927) and 0.9923 (95% CI: 0.9905 to 0.9941), respectively. Additionally, we also performed model validation on a hand-held CFP dataset (i.e., Hand-Held) with lower resolution compared with conventional CFP, containing 6 fundus conditions: normal (N), DR, tessellation (TSLN), drusen (DN), myopia (MYA), and others (O). RetExpert achieved better performance with a mean AUROC of 0.8621, a mean AUPRC of 0.6316 and a kappa of 0.4606, demonstrating significantly better generalizability than other ophthalmic FMs. Notably, RetExpert’s higher *C*-score compared to FLAIR reflects more confused predictions when processing hand-held fundus images containing an interfering factor.

**Table 2:** Unseen private clinical datasets for validating baseline FMs and our RetExpert model

Test on STDR	F1 score	Kappa	*C*-score	AUPRC	AUROC	*P*-value
FLAIR	0.6667	0.0893	0.4822	0.6704 ± 0.0126	0.6223 ± 0.0101	<0.001
VisionFM	0.6667	0.3061	0.3238	0.8370 ± 0.0078	0.7746 ± 0.0083	<0.001
RETFound	0.6985	0.4110	0.3530	0.8788 ± 0.0065	0.8364 ± 0.0070	<0.001
RetExpert	**0.7306**	**0.4647**	**0.2859**	**0.8936** ± **0.0062**	**0.8572** ± **0.0067**	Ref.
Test on Vietnam	F1 score	Kappa	*C*-score	AUPRC	AUROC	*P*-value
FLAIR	0.6667	0.2310	0.1762	0.5142 ± 0.0463	0.7340 ± 0.0301	<0.001
VisionFM	0.6667	0.2193	0.3444	0.4895 ± 0.0509	0.7521 ± 0.0283	<0.001
RETFound	0.6667	0.1890	0.4783	0.5714 ± 0.0526	0.8159 ± 0.0251	<0.001
RetExpert	**0.7842**	**0.3512**	**0.1365**	**0.6278** ± **0.0449**	**0.8477** ± **0.0120**	Ref.
Test on AITS	F1 score	Kappa	*C*-score	AUPRC	AUROC	*P*-value
FLAIR	0.6667	0.0151	0.6313	0.1185 ± 0.0151	0.4822 ± 0.0262	<0.001
VisionFM	0.6667	0.0125	1.4695	0.1185 ± 0.0151	0.4673 ± 0.0262	<0.001
RETFound	0.7374	0.2473	0.5047	0.4964 ± 0.0444	0.8066 ± 0.0220	0.043
RetExpert	**0.8087**	**0.3400**	**0.3871**	**0.5590** ± **0.0425**	**0.8417** ± **0.0193**	Ref.
Test on CUHK-GON(D)	F1 score	Kappa	*C*-score	AUPRC	AUROC	*P*-value
FLAIR	0.5156	0.2111	<0.0001	0.8628 ± 0.0082	0.4337 ± 0.0136	<0.001
VisionFM	0.5723	0.2707	<0.0001	0.8877 ± 0.0068	0.5133 ± 0.0133	<0.001
RETFound	0.8385	0.6830	<0.0001	0.9725 ± 0.0025	0.8571 ± 0.0096	<0.001
RetExpert	**0.9298**	**0.8601**	<0.0001	**0.9916** ± **0.0011**	**0.9547** ± **0.0055**	Ref.
Test on CUHK-GON(M)	F1 score	Kappa	*C*-score	AUPRC	AUROC	*P*-value
FLAIR	0.6017	0.3060	<0.0001	0.8963 ± 0.0112	0.5486 ± 0.0235	<0.001
VisionFM	0.4494	0.1455	<0.0001	0.9012 ± 0.0107	0.5652 ± 0.0233	<0.001
RETFound	0.7694	0.5576	<0.0001	0.9628 ± 0.0052	0.8138 ± 0.0182	<0.001
RetExpert	**0.9249**	**0.8501**	<0.0001	**0.9923** ± **0.0018**	**0.9594** ± **0.0088**	Ref.
Test on Hand-Held	F1 score	Kappa	*C*-score	AUPRC	AUROC	*P*-value
FLAIR	0.3612	0.2211	**0.1022**	0.3733 ± 0.2087	0.6999 ± 0.0884	<0.001
VisionFM	0.4622	0.3465	0.3776	0.5161 ± 0.1715	0.8321 ± 0.0833	<0.001
RETFound	0.5342	0.4101	0.3550	0.5438 ± 0.2153	0.8182 ± 0.1074	<0.001
RetExpert	**0.5646**	**0.4606**	0.1317	**0.6316** ± **0.1382**	**0.8621** ± **0.0933**	Ref.

A detailed description of these datasets is provided in Supplementary Table 3.

Bold values indicate the best performance.

## Discussion

This study proposed RetExpert, a clinically translatable FM-based model for simultaneous multi-disease detection in CFPs, validated across diverse datasets collected from real-world clinical environments. The Sankey diagram in Supplementary Fig. 1 visualizes the predictions of our RetExpert on the concurrent multi-disease MuReD and ODIR datasets, showcasing its state-of-the-art performance in fundus multi-disease detection. Previous studies have shown that ophthalmic FMs offer no significant advantages over traditional ImageNet-based pre-trained models in certain downstream tasks^24^. However, our proposed RetExpert leveraged ophthalmic FMs, optimized through a clinical problem-oriented strategy, to demonstrate comprehensive superiority. RetExpert outperformed not only non-FM-based models but also related ophthalmic FMs in multi-disease detection, demonstrating particularly notable gains on various unseen OOD fundus datasets. Our study reinforces confidence in the clinical applicability of the ophthalmic FM-based method in real-world clinical settings.

To fully harness the clinical utility of the pre-trained ophthalmic FM in real-world clinical settings and effectively leverage its generalizable foundational knowledge, this study constructed a series of adapter-based knowledge units (i.e., AKUs) based on the blocks of RETFound for fundus multi-disease detection in CFPs. During model training, freezing the knowledge units of the FM while fine-tuning the adapter of each AKU constituted a prudent strategy, offering two key advantages: first, it preserves the pre-learned knowledge structure; second, it drastically reduces resource consumption (training parameters are reduced from 303.22 to 6.45 M), lowering the resource requirements for model development without compromising performance. Notably, different adapter-based training strategies are closely correlated with model performance, necessitating meticulous design and thorough analysis. Meanwhile, an excessively small number of trainable parameters is prone to overfitting. To alleviate this issue and enhance the generalizability of RetExpert, we proposed a novel SOA module as a compensatory mechanism. In each training iteration, one AKU was randomly selected to align its features with those of the final AKU, thereby enhancing the generalizability of each individual AKU and, consequently, the overall generalizability of RetExpert. Our proposed adapter-based architecture, integrated with the SOA module, underscores RetExpert’s inherent knowledge integrity and enhance its generalizability in cross-domain fundus disease detection tasks.

To bridge the translational gap in multi-disease detection models, it is essential to give greater consideration on imbalanced data distribution, multi-disease uncertainty, and inter-disease confusion. In this study, beyond addressing the imbalanced or long-tailed distribution of disease data via the existing RAL method, we incorporated two further critical considerations: first, the multi-disease uncertainty in fundus multi-disease detection (by proposing UAML), and second, the inter-disease confusion of the fundus multi-disease detection model (by proposing FDCM and *C*-score). Regarding the first challenge, conventional uncertainty estimation typically operates on the model’s overall output, which is less rational for fundus multi-disease detection models. In real-world clinical diagnosis, ophthalmologists exhibit varying levels of expertise across different fundus diseases, with strengths and weaknesses specific to each condition. Therefore, uncertainty should be estimated separately for each disease-specific classification head in the model, as this reflects the model’s true capability in detecting individual fundus diseases. Thus, the proposed UAML within RetExpert holds substantial clinical significance. For the second challenge, existing fundus multi-disease detection models frequently overlooked the correlations between disease labels, often leading to inter-disease confusion. Prior to model training, we proposed a FDCM to quantify the relative similarity between any two diseases, enabling RetExpert to explicitly incorporate disease co-occurrence relationships during training and thereby mitigate confusion between disease pairs. Additionally, the proposed *C*-score serves as a metric to evaluate the model’s inter-disease confusion degree. Under comparable detection performance, *C*-score reflects model reliability, providing better decision support for healthcare professionals in fundus multi-disease detection tasks.

Clinical domain shift is another critical challenge that dictates the applicability of multi-disease detection models. To enhance its clinical utility, our RetExpert introduced a novel module-wise TTA method (TTUL + TTPL). Notably, RetExpert was not designed to perform instantaneous per-image adaptation or streaming updates. Instead, the proposed TTUL + TTPL method was a lightweight TTA process. During inference, the adapter and normalization parameters were rapidly updated using target-domain test samples during, eliminating the need for full model retraining. While the Algorithm in Supplementary Note 2 illustrates that the framework supports multiple forward–backward optimization iterations over batches of test data, our experiments have proved that performance improvement also could be achieved with just a single forward–backward iterations. Thus, RetExpert improved detection performance without a significant increase in computational overhead or inference time, as detailed in Supplementary Table 1. Additionally, the integration of TTUL and TTPL supports sequential processing (batch size = 1) of CFPs, making it well-suited for the real-world clinical workflow. Crucially, RetExpert’s parameters are not accumulated across different patients, thereby ensuring the effective preservation of the RetExpert’s originally learned knowledge for the clinical downstream task.

Our RetExpert still has several limitations that warrant further investigation. As RetExpert is built upon ophthalmic FMs, its performance is inherently dependent on the capabilities of the base FM. This study did not explore the performance gains achievable by RetExpert with each specific FM; instead, it proposes an FM-based AKU framework for researchers to utilize in their specific tasks. Moreover, RetExpert currently requires GPU-based inference. Although our proposed TTUL + TTPL enhanced RetExpert’s performance, it also increased inference latency, posing challenges for real-time clinical processing. While RetExpert achieved a speed exceeding 3 FPS on the GPU device, its latency would increase significantly if deployed on edge computing devices, making real-time response (a critical clinical requirement) currently unattainable. Furthermore, although RetExpert offered flexible parameter adaptability, it was not a continual learning system and could not incrementally accumulate knowledge from new data. Additionally, how to effectively control model parameters during testing to mitigate regulatory and irreproducibility risks remains an open research question. These limitations highlight promising directions for future model optimization.

In conclusion, we propose RetExpert, a clinically adaptive model that enhances ophthalmic FMs for robust multi-disease detection from CFPs. RetExpert integrates an adapter-based SOA module into the knowledge units of FMs, effectively improving its generalizability. To address key challenges in multi-disease recognition, including data imbalance, multi-disease uncertainty, and inter-disease confusion, RetExpert incorporates a long-tail-aware loss (RAL), a newly refined multi-disease uncertainty method (UAML), and a novel FDCM as prior knowledge. Importantly, during inference, RetExpert innovatively introduces a TTA method combining TTUL and TTPL to mitigate domain shift in real-world clinical settings. Extensive evaluations on 15 public and private unseen clinical datasets demonstrate that RetExpert surpasses state-of-the-art ophthalmic FMs in multi-disease detection performance, reliability, and adaptability. Our work provides a clinically feasible and translation-oriented solution for automated multi-disease screening from CFPs, laying the foundation for more reliable AI-assisted diagnosis in ophthalmology.

## Methods

**Fig. 10:**
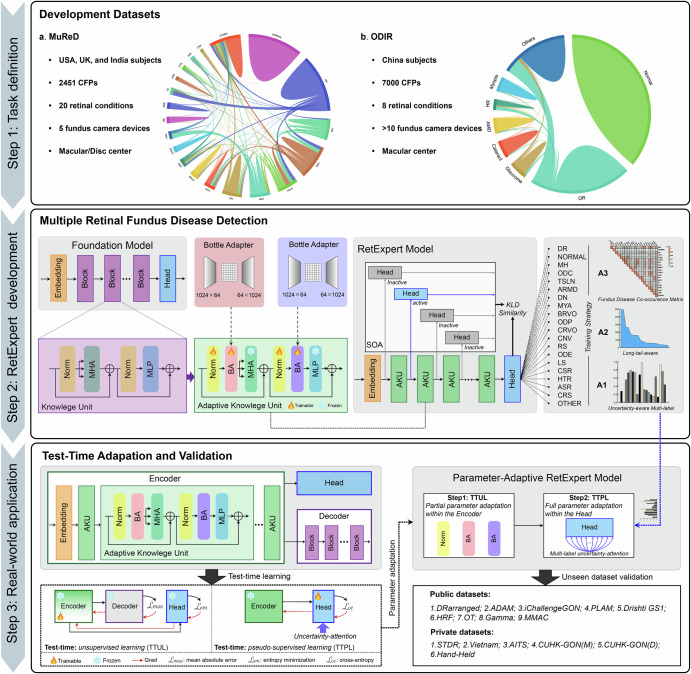
The proposed RetExpert development framework. This framework comprises three steps: task definition, RetExpert development, and real-world application. The workflow is delineated as follows: *Step 1* defines the specific tasks for fundus multi-disease detection in CFPs. *Step 2* leverages adaptive knowledge units (AKUs) from ophthalmic FMs to construct a specialized RetExpert model architecture with several training strategies, which addresses two key challenges: (1) model generalizability; (2) data imbalance, model uncertainty, and inter-disease confusion in multi-disease recognition. *Step 3* introduces a test-time adaptation (TTA) method within RetExpert that utilizes the multi-disease uncertainty outputs for parameter adaptation, addressing another challenge: (3) domain shift across diverse clinical scenarios.

This study is motivated by both the prevalence of co-occurring fundus diseases and the unmet need for robust models for multi-disease detection under real-world clinical variability. Therefore, we proposed a novel RetExpert, which was evolved from a general ophthalmic FM into a specialized model for detecting multiple fundus diseases. Our RetExpert consists of three core stages: task definition, RetExpert development, and real-world application, with detailed methodology illustrated in Fig. 10. RetExpert has integrated innovative solutions designed to address the key challenges of developing and deploying AI in real-world scenarios, which are elaborated in the following sections.

### Multi-disease fundus datasets for RetExpert development

Two open-access multiple co-occurring fundus disease datasets, MuReD^7^ and ODIR^37^, were employed in the development of our RetExpert model. These datasets encompass a wide range of abnormal categories commonly encountered in clinical practice, simulating the complex spectrum of concurrent fundus diseases observed in real-world scenarios. Concretely, the MuReD dataset encompasses 20 categories of fundus conditions, including an “other” category for cases outside the predefined classifications. Each category contains a minimum of 30 samples. Similarly, the ODIR dataset is a structured ophthalmic database comprising images from 5000 patients, including demographic information such as age and gender, as well as CFPs of both left and right eyes. The annotations were performed by trained human readers under strict quality control protocols, classifying CFPs into eight categories, including one labeled “other”. The fundus disease distributions of MuReD and ODIR are presented in the task definition panel of Fig. 10.

### Unseen clinical datasets for RetExpert validation

A comprehensive collection of both publicly available and private datasets comprising real-world CFPs was assembled to validate the effectiveness of our proposed RetExpert. The inclusion criterion was a sample size exceeding 30. Consequently, our study incorporated nine distinct public datasets: DRarranged^38^, ADAM^39^, iChallengeGON^40^, PALM^41^, Drishti-GS^42^, HRF^43^, GAMMA^44^, MMAC^45^, and OTFID^46^. In addition, six private datasets were included: the Sight-Threatening Diabetic Retinopathy (STDR) study from the Chinese University of Hong Kong (CUHK)^47^, the AI triage-screening (AITS) study from the CUHK Eye Center^48^, a DR study from the Binh Dinh Eye Hospital (BDEH) in Vietnam (Vietnam)^49^, the glaucomatous optic neuropathy (GON) study from the CUHK Eye Center (CUHK-GON(D) and CUHK-CON(M))^50^, and the hand-held multiple fundus disease study from the Beijing University of Technology (Hand-Held) to further validate our method. Detailed data descriptions of both public and private datasets, including disease category, data volume, camera type, region, age, field of view (FOV), image resolution, and patient-level overlap, can be seen in Supplementary Table 2 and Table 2. The acquisition and utilization of all datasets were conducted in strict adherence to ethical standards and regulatory requirements.

### Model architecture of RetExpert

Previous ophthalmic FMs primarily adopted the ViT architecture^30,51^, which consists of multiple cascaded blocks. We conceptualized these blocks as knowledge units, designed to extract multi-level and multi-scale features. Knowledge units learned from large-scale datasets were capable of effectively adapting to diverse clinical tasks. Our proposed model, RetExpert, was constructed on the knowledge units derived from RETFound^18^. To preserve the integrity of the pre-trained knowledge structure, we inserted lightweight bottle adapters^52^ into each knowledge unit, with only the normalization layers and the resulting AKUs being trainable. More importantly, to enhance generalizability across all AKUs, we innovatively proposed a SOA module. The SOA regularizes the model by aligning the features from an intermediate AKU with those of the final AKU, as described in Eq. (1). Specifically, we calculated and minimized the Kullback-Leibler Divergence (KLD)^53^ between the features of ${I}^{{th}}$ output ${f}_{{AKU}}^{I}$ and the final output ${f}_{{AKU}}^{K}$ during each iterative learning step of the model training process, thereby strengthening adapters’ feature representation generalizability and the task-specific relevance of each AKU. Inside, ${f}_{{AKU}}^{I}$ is derived from Eqs. (2) and (3). Concretely, the variable $I$ in Eq. (3) follows the uniform distribution $U\{1,\,2,\ldots ,K-1\}$, and in each iteration, a discrete value between $1$ and $K-1$ is randomly selected to construct one-hot vector ${V}^{(I)}$, where $K$ is the total number of AKUs. This stochastic nature of this selection process additionally helps mitigate the risk of overfitting across each adapter within the AKU, thereby enhancing the overall generalizability of RetExpert.

1 $${{\mathcal{L}}}_{{KLD}}={D}_{{KL}}({f}_{{AKU}}^{K}||{f}_{{AKU}}^{I})={E}_{{x \sim f}_{AKU}^{K}}\left[{\mathrm{log}}{f}_{{AKU}}^{K}\left(x\right)-{\mathrm{log}}{f}_{{AKU}}^{I}\left(x\right)\right]$$

2 $${f}_{{AKU}}^{I}=\left({f}_{{AKU}}^{1},\,{f}_{{AKU}}^{2},\ldots ,{f}_{{AKU}}^{K-1}\right)\cdot {V}^{(I)}$$

3 $${V}^{(I)}={({v}_{1},\,{v}_{2},\ldots ,{v}_{K-1})}^{T},\,{v}_{i}=\left\{\begin{array}{l}1\,i=I\\ 0\,i\,\ne \,I\end{array}\right.$$

### Training strategy for RetExpert

Long-tail-aware and uncertainty-aware learning. Fundus diseases exhibit a long-tailed distribution, leading to a significant imbalance in data distribution between common and rare conditions in training datasets. We applied the Robust Asymmetric Loss (RAL)^54^ to facilitate long-tailed-aware learning, addressing the challenges of sparse and imbalanced multi-label classification. Moreover, inspired by the uncertainty estimation framework (UIOS)^55^, we proposed an improved uncertainty quantification in multi-label classification tasks, designated as UAML. We hypothesize that, similar to human experts who demonstrate varying diagnostic proficiency across different diseases, the model should also exhibit disease-specific confidence levels rather than maintaining a uniform uncertainty score. Therefore, unlike the UIOS approach that estimated a single uncertainty score for the entire model output, our proposed UAML module generated distinct uncertainty scores for each disease-specific classification head. The complete algorithmic details can be seen in Supplementary Note 1.

Medical prior knowledge learning. Multi-disease co-occurrence is a common clinical manifestation in fundus disorders. To enable RetExpert to capture the intrinsic relationships between fundus diseases and reduce inter-disease confusion, we innovatively constructed a FDCM, informed by both ophthalmologist-annotated pathological relationships and statistical co-occurrence patterns^7,56^. The FDCM as the medical prior knowledge was subsequently used to guide the training of RetExpert. For any two fundus diseases, we define five levels of co-occurrence coefficients, each with specific definitions as detailed in Table 3. Notably, the “complete” level represents the self-co-occurrence coefficient of a given disease, represented by the diagonal elements of the FDCM. For the “minimal” level, a zero probability was assigned to disease pairs considered clinically implausible. The FDCMs of MuReD and ODIR datasets can be seen in Supplementary Fig. 2. The loss function for learning the FDCM is defined as follows:

5 $${{\mathcal{L}}}_{{FDCM}}=\frac{1}{N}\mathop{\sum }\limits_{n=1}^{N}\mathop{\sum }\limits_{1\le i\le j}^{C}\left|\begin{array}{c}{\left({p}_{i}-{p}_{j}\right)}^{2}-{\left(1-{m}_{ij}\right)}^{2}\end{array}\right|$$

where *p*_*i*_ and *p*_*j*_ denote the output probabilities for disease *i* and *j*, respectively; *m*_*ij*_ represents the co-occurrence coefficient between disease *i* and *j* in the FDCM; *C* is the total number of disease categories; and *N* corresponds to the number of training samples in the dataset.

**Table 3:** Definition of fundus disease co-occurrence matrix (FDCM)

Co-occurrence level	Description	Coefficient
Complete	Two categories are the same.	1.00
High	Two categories share common fundus features, with a high likelihood of co-occurrence.	0.75
Moderate	Two categories exhibit similar fundus features, with a potential to co-occur.	0.50
Low	Two categories have distinct fundus features, with a low probability of co-occurrence.	0.25
Minimal	Two categories are highly unlikely to co-occur or exist within the same anatomical location.	0.00

### Test-time adaptation of RetExpert

Well-trained multi-disease detection models should demonstrate robustness against domain shift in variable clinical settings when deployed, particularly regarding differences in pathologies, patient demographics, and imaging protocols. Thus, during inference, RetExpert further introduced a module-wise test-time adaptation (TTA) framework that integrates both Test-Time Unsupervised Learning (TTUL) and Test-Time Pseudo-supervised Learning (TTPL). This integration enables the dynamic fine-tuning of the pre-trained RetExpert parameters based on unseen fundus images at test time. The details are illustrated in the clinical application of RetExpert (Fig. 10). Specifically, during the TTUL phase, the task-specific head was kept frozen, while only the adapters and normalization layers in the encoder were adapted via self-supervised learning. This process was optimized with a combined loss of mean squared error (MSE) and soft entropy (SE) over *K* iterations. In the subsequent TTPL phase, the encoder was frozen, and the task-specific head was adapted. The predicted probabilities were binarized into pseudo-labels, which were then used alongside the multi-disease uncertainty scores from the UAML module as attention weights to guide the optimization of the task-specific head during the *I*-step supervised learning. In our study, to mitigate significant computational latency, we set *K* and *I* to 1 across our experiments during TTA phase. For each test batch, RetExpert adapts its parameters using either a single fundus image or a group of images and then resets its own parameters to their prior state upon completion of the inference task. Consequently, this design prevents cumulative drift in the RetExpert model’s parameters over time. Moreover, since no stochastic operations are involved during inference and a unified optimization formulation is applied, RetExpert guarantees consistent outputs for identical fundus image(s) input. This design preserves the pre-acquired knowledge of the RetExpert model while permitting targeted adaptation of its parameters to truly unseen datasets during test-time inference. A detailed description of our algorithm is provided in Supplementary Note 2.

### Evaluation metrics and implementation

Evaluation metrics. We evaluated our RetExpert and comparative models using a comprehensive set of standard metrics, including accuracy, precision, recall, F1 score, area under the precision-recall curve (AUPRC), area under the receiver operating characteristic curve (AUROC), and Cohen’s Kappa coefficient. Beyond these conventional metrics, we also proposed a novel evaluation metric, termed the confusion score (*C*-score), as defined by Eq. (6). *C*-score quantifies the degree of confusion between pairs of fundus diseases learned by a model, providing clinicians with a practical inter-disease confusion assessment metric for fundus multi-disease models in real clinical settings. In Eq. (6), *µ*_*a/b*_, *S*_*a/b*_ represents the two-dimensional mean and variance of disease categories *a* and *b*, respectively.

6 $$C-{score}=\frac{\sqrt{{S}_{a}\cdot {S}_{b}}}{\left|{\mu }_{a}-{\mu }_{b}\right|}$$

Statistical Analysis. For experiments involving repeated runs, all trials were conducted with five independent random seeds. The reported performance metrics were presented as the mean with 95% confidence interval (CI) calculated from these five replicates using a t-distribution. The corresponding *P*-values were also derived from a paired t-test where applicable, to assess the statistical significance of the observed differences. For the evaluation on the unseen clinical datasets, where a single model was evaluated once on each test dataset, the 95% CIs and *P*-values for the AUROC and AUPRC metrics were computed using the DeLong test^57^.

Implementation details. All experiments were conducted on a single high-performance NVIDIA A100 GPU with 40 GB of VRAM using the PyTorch deep learning framework. The final configuration employed the AdamW optimizer with an initial learning rate of 1e-3 and a weight decay of 5e-2, which was smoothly reduced with each training iteration. We used a batch size of 16 and trained for a total of 200 epochs. The architecture of RetExpert yielded 309.67 M total parameters (6.45 M trainable) and a model size of 1.15 GB, representing an approximately 2.2% increase over RETFound. All comparative models in this study were trained under identical configurations, including batch size, number of epochs, and optimizer settings. To enhance transparency and reproducibility, the implementation details are partially provided in our public code repository, available at: https://github.com/OVS-AILab/RetExpert.

## Supplementary information

Supplementary Information

## Data Availability

The public datasets utilized in this study are available through the following links: MuReD: https://www.kaggle.com/datasets/abhirampolisetti/multi-label-retinal-disease-mured-dataset ODIR: https://odir2019.grand-challenge.org/dataset/ ADAM: https://amd.grand-challenge.org/ DRarranged: https://www.kaggle.com/datasets/amanneo/diabetic-retinopathy-resized-arranged/data Drishti-GS: http://cvit.iiit.ac.in/projects/mip/drishti-gs/mip-dataset2/Dataset_description.php GAMMA: https://aistudio.baidu.com/competition/detail/90/0/introduction iChallengeGON: https://aistudio.baidu.com/datasetdetail/177198 MMAC: https://codalab.lisn.upsaclay.fr/competitions/12441#learn_the_details-ranking PALM: https://palm.grand-challenge.org/ OTFID: https://zenodo.org/records/5156953#.YbqGF2jMKUk HRF: https://www5.cs.fau.de/research/data/fundus-images/.

## Code availability

Code available at https://github.com/OVS-AILab/RetExpert.
