# Supplementary material for: A test-time clinically adaptive framework for detecting multiple fundus diseases harnessing ophthalmic foundation models
Source: NPJ Digit Med. 2026 Mar 2;9:300. doi: 10.1038/s41746-026-02480-1 (PMC13065778; doi:10.1038/s41746-026-02480-1)
Supplement: Supplementary file 1 — Supplementary Information [file 41746_2026_2480_MOESM1_ESM.pdf]

Supplementary Information

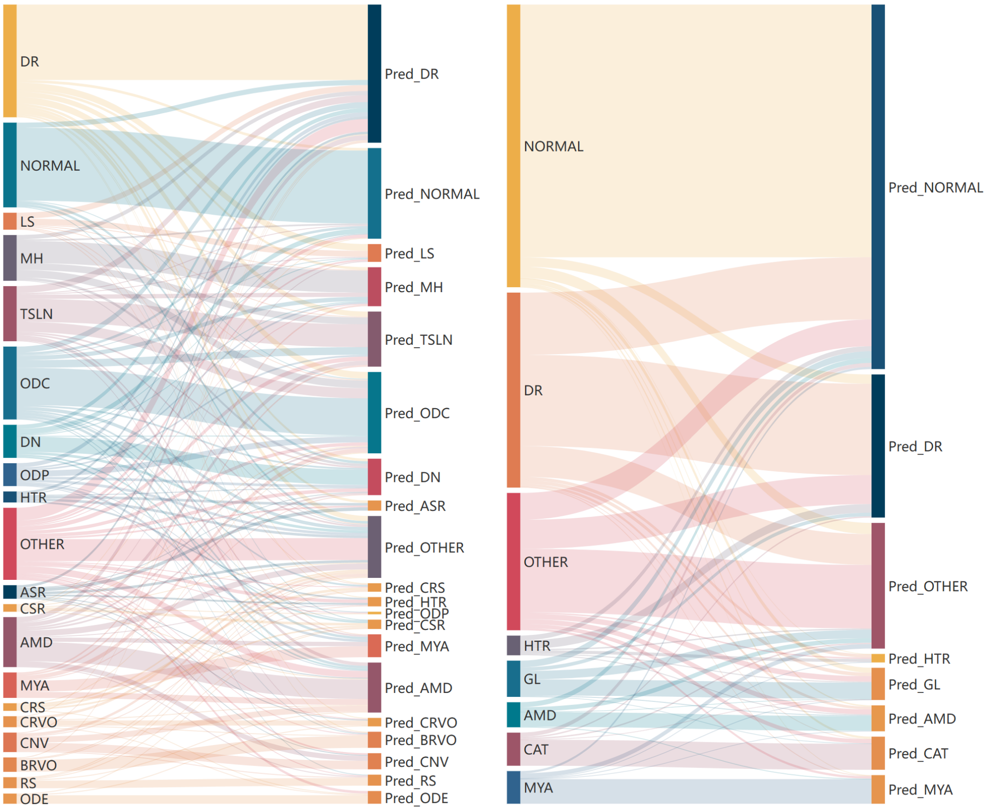

Supplementary Fig. 1: Sankey diagrams illustrating label mapping between ground truth and RetExpert predictions. Diagrams are presented for two multi-label fundus image datasets: MuReD (left panel) and ODIR (right panel). In each diagram, ground-truth labels (left nodes) are mapped to the model's predicted labels (right nodes). The width of a flow represents the number of samples, highlighting predominant correct predictions and major misclassification pathways across disease categories.

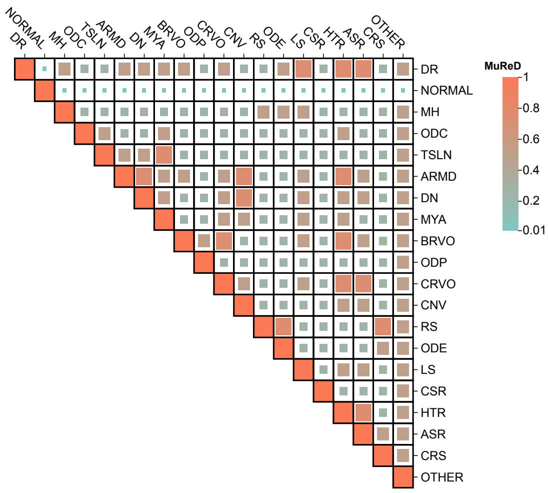

(a)

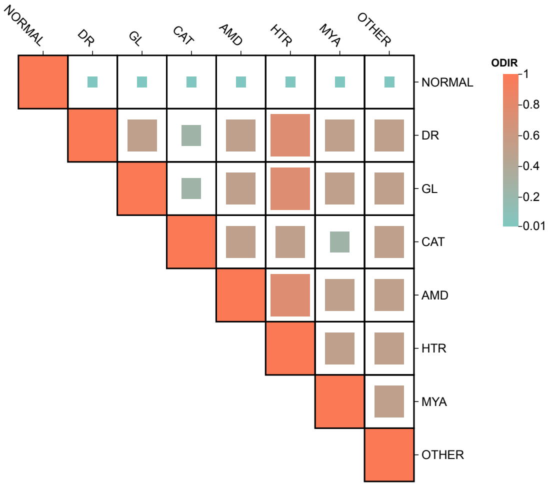

(b)

Supplementary Fig. 2: Normalized FDCM for multi-label fundus image datasets: (a) MuReD and (b) ODIR. Each entry at row i, column j indicates the relative frequency of co-occurrence of the two diseases in the same fundus image. High co-occurrence coefficients reflect common comorbid presentations, whereas near-zero values denote mutually exclusive conditions. These matrices were integrated into Ret-Expert during training to capture inter-label dependencies and reduce inter-disease confusion.

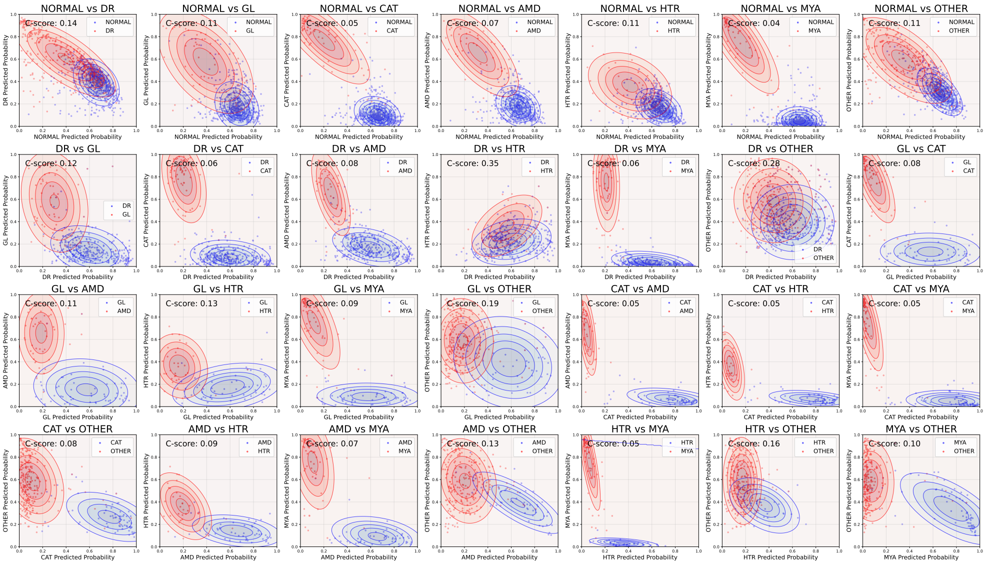

Supplementary Fig. 3: Probability scatter plots of the C-score for RetExpert's predictions on the ODIR dataset (without FDCM).

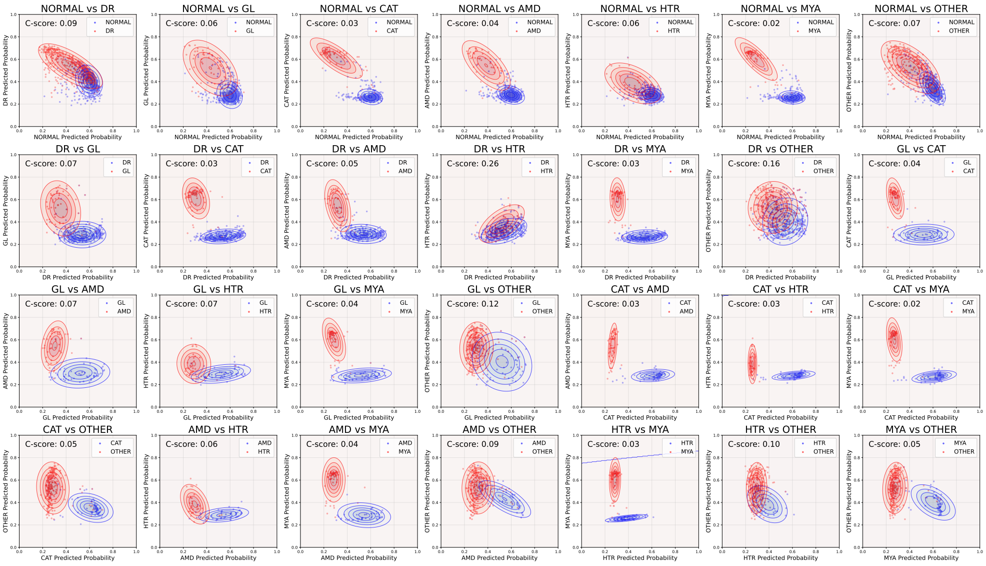

Supplementary Fig. 4: Probability scatter plots of the C-score for RetExpert's predictions on the ODIR dataset (with FDCM).

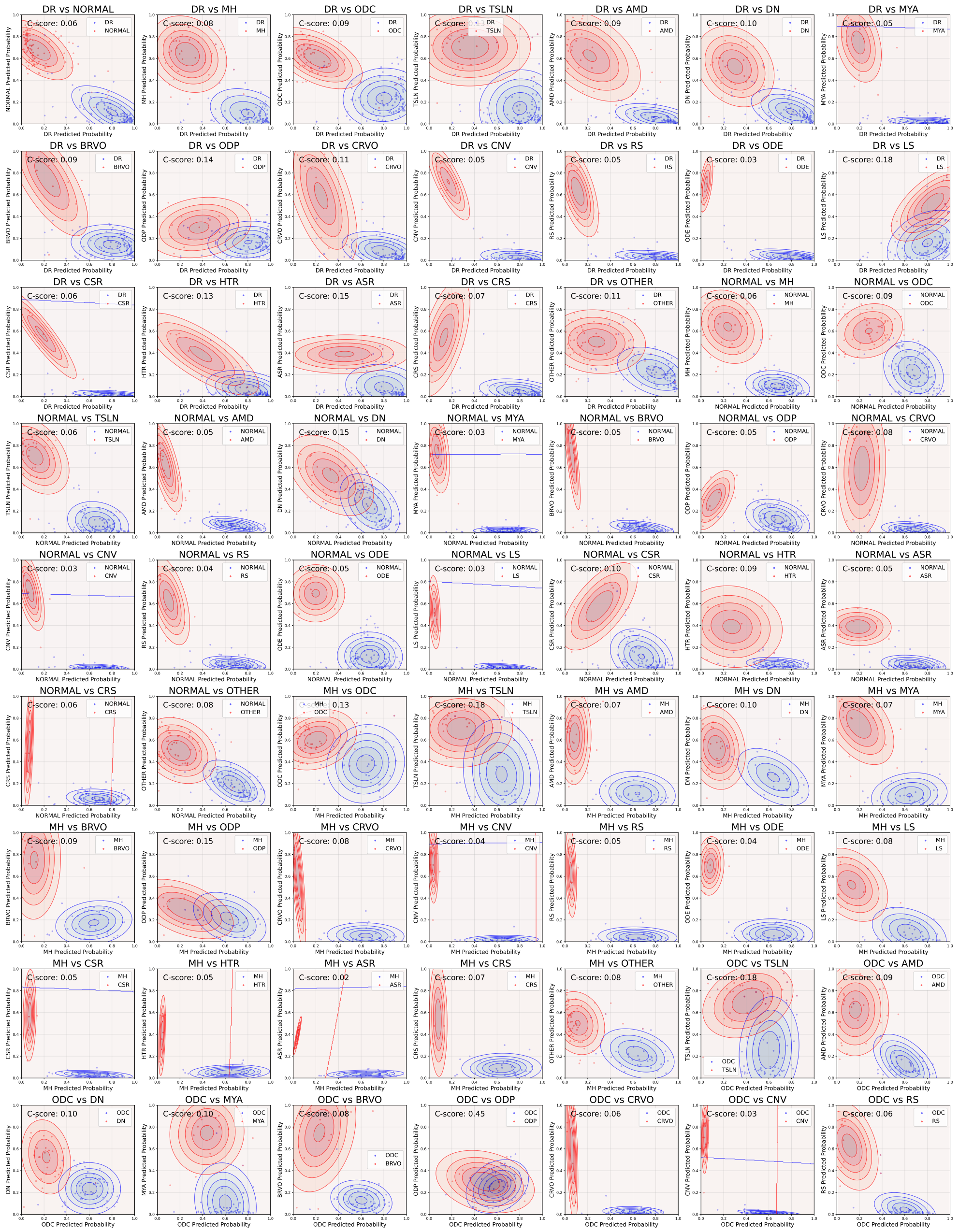

Supplementary Fig. 5: Probability scatter plots of the C-score for RetExpert's predictions on the MuReD dataset (without FDCM).(part 1/3)

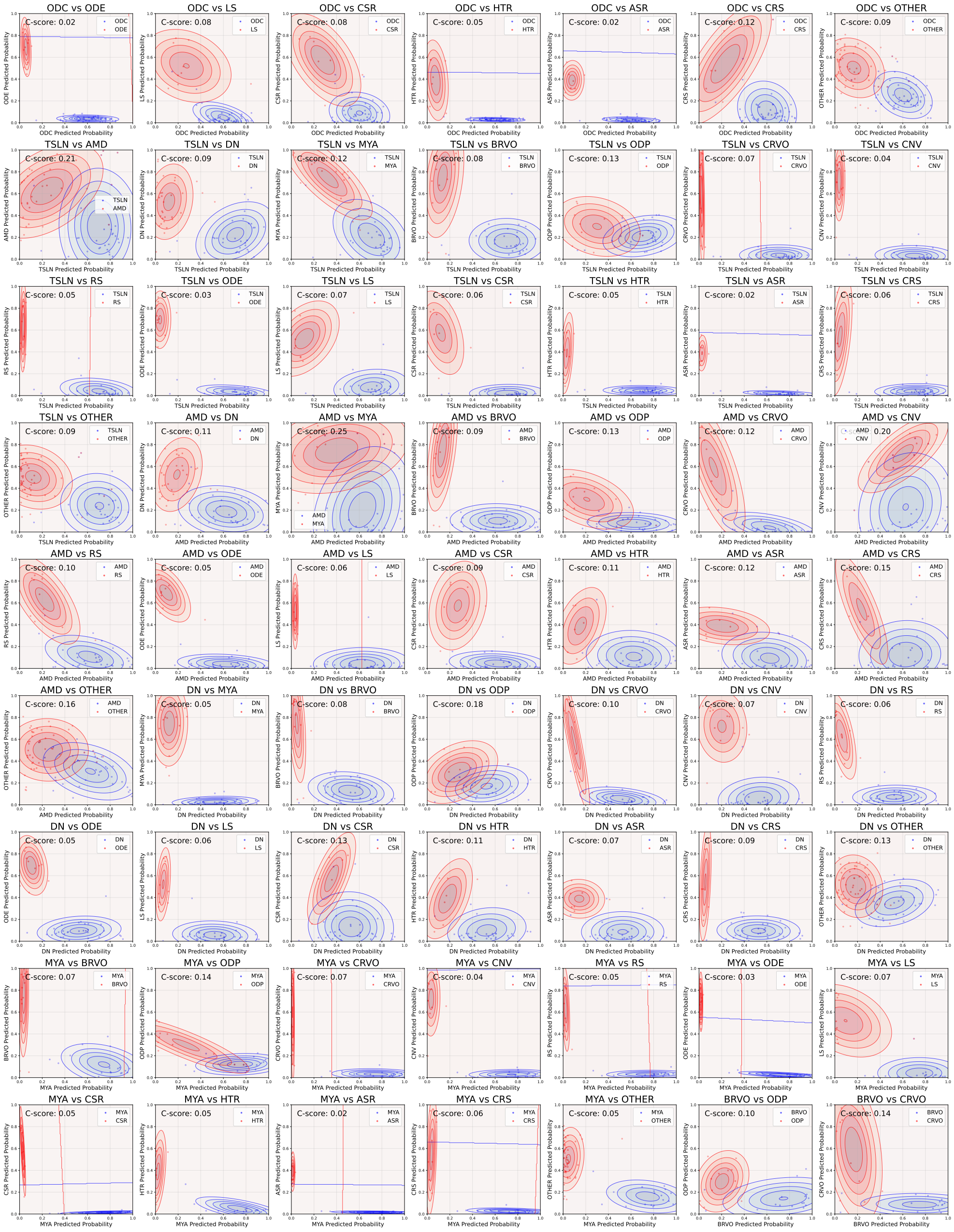

Supplementary Fig. 6: Probability scatter plots of the C-score for RetExpert's predictions on the MuReD dataset (without FDCM).(part 2/3)

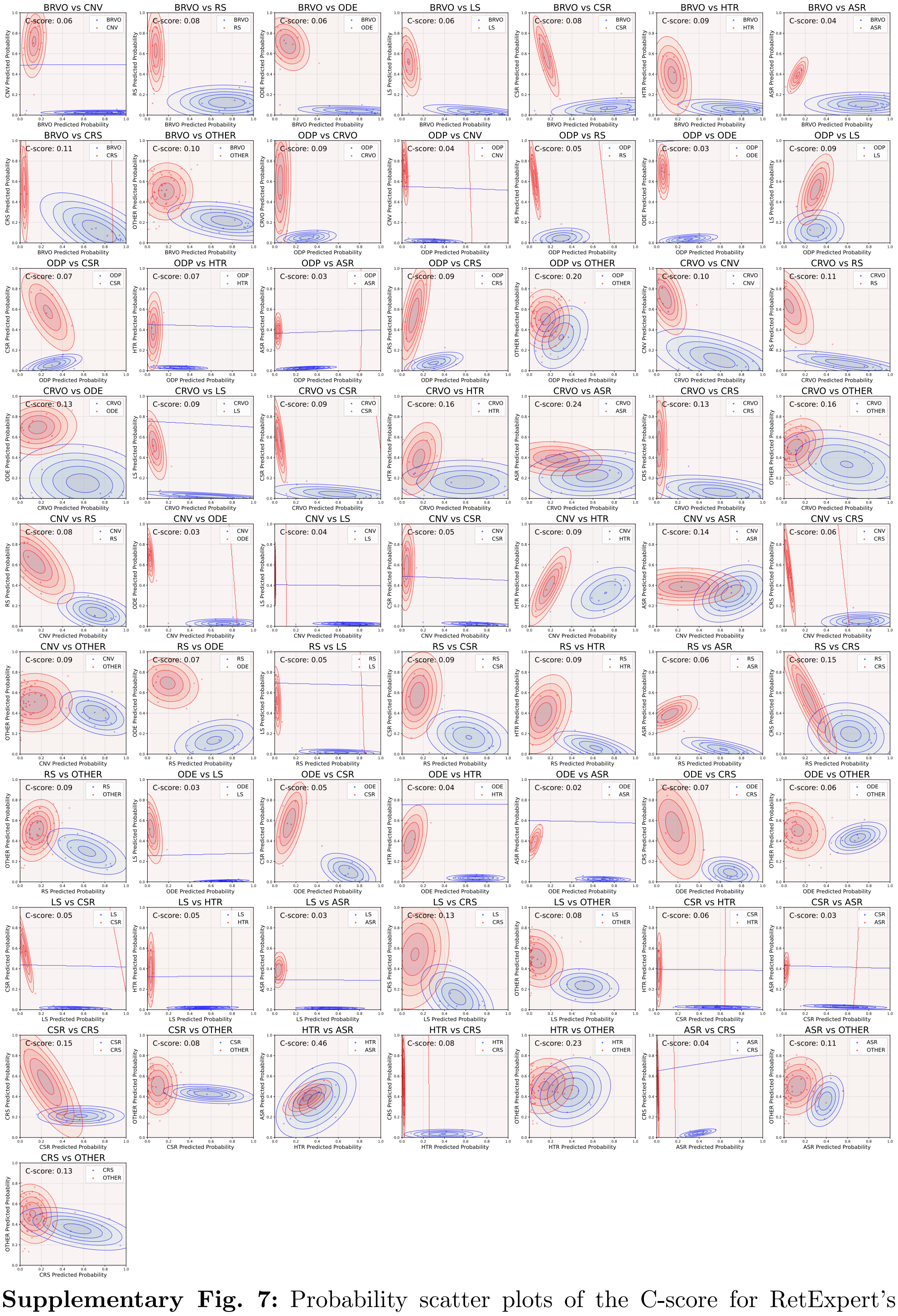

Supplementary Fig. 7: Probability scatter plots of the C-score for RetExpert's predictions on the MuReD dataset (without FDCM).(part 3/3)

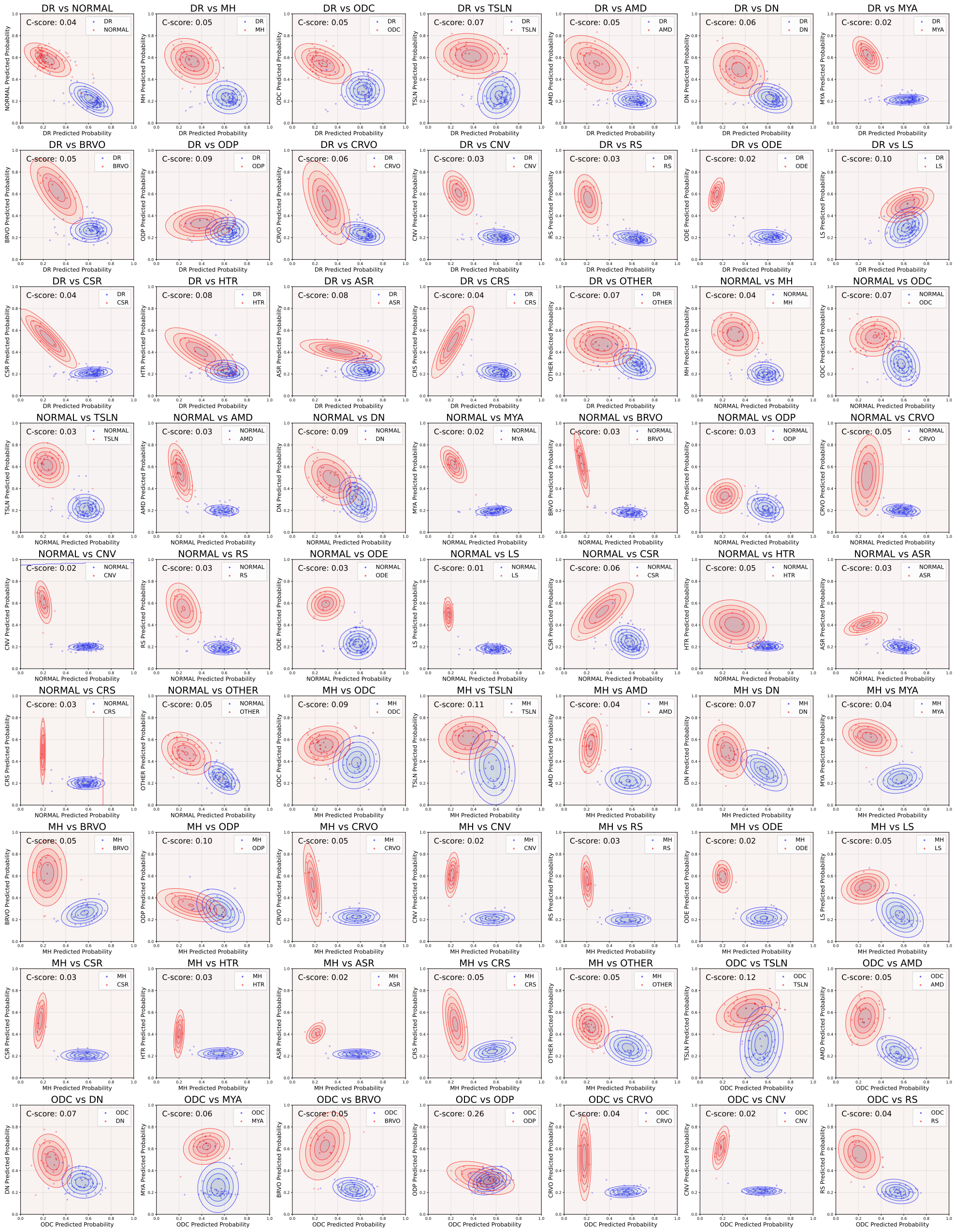

Supplementary Fig. 8: Probability scatter plots of the C-score for RetExpert's predictions on the MuReD dataset (with FDCM).(part 1/3)

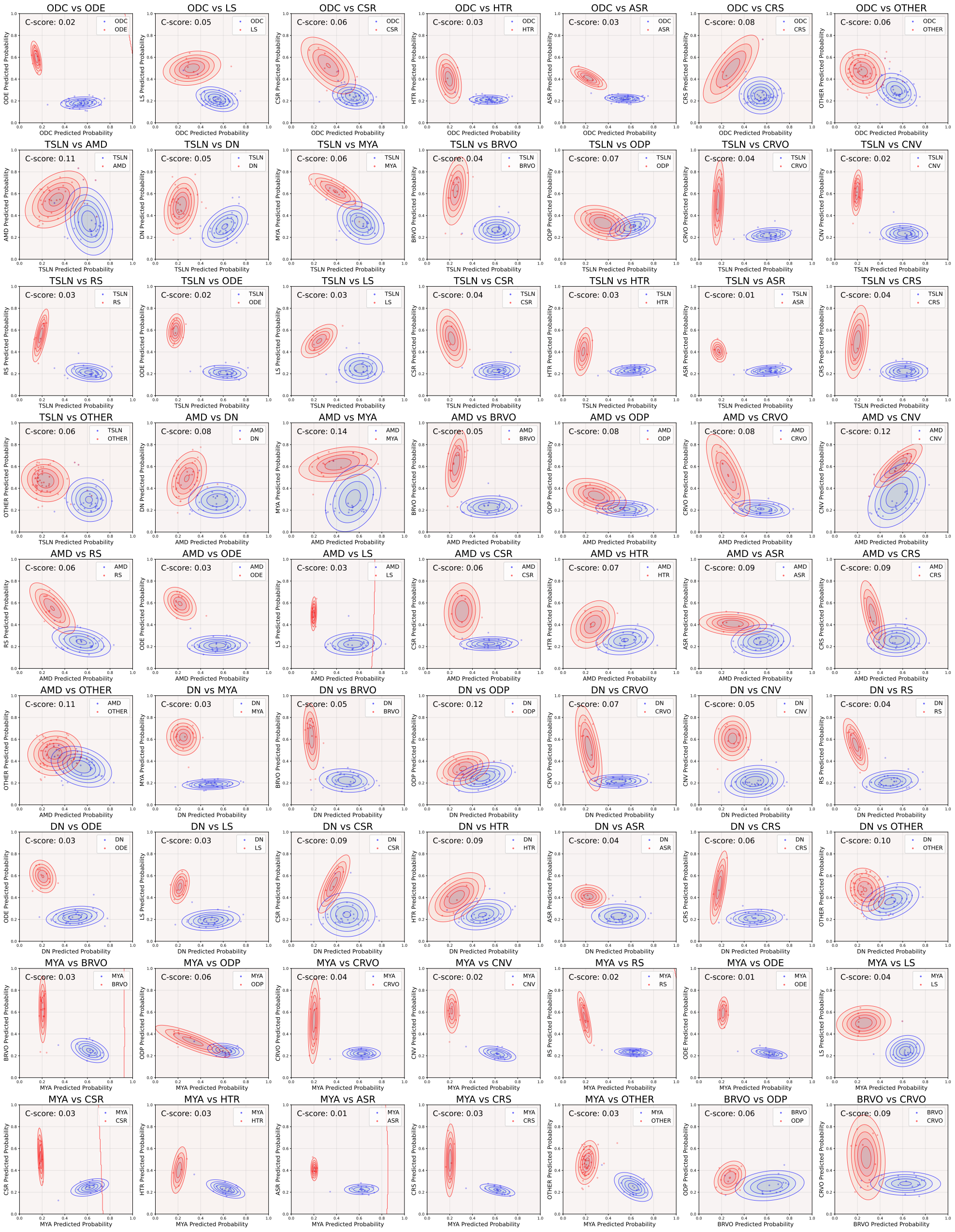

Supplementary Fig. 9: Probability scatter plots of the C-score for RetExpert's predictions on the MuReD dataset (with FDCM).(part 2/3)

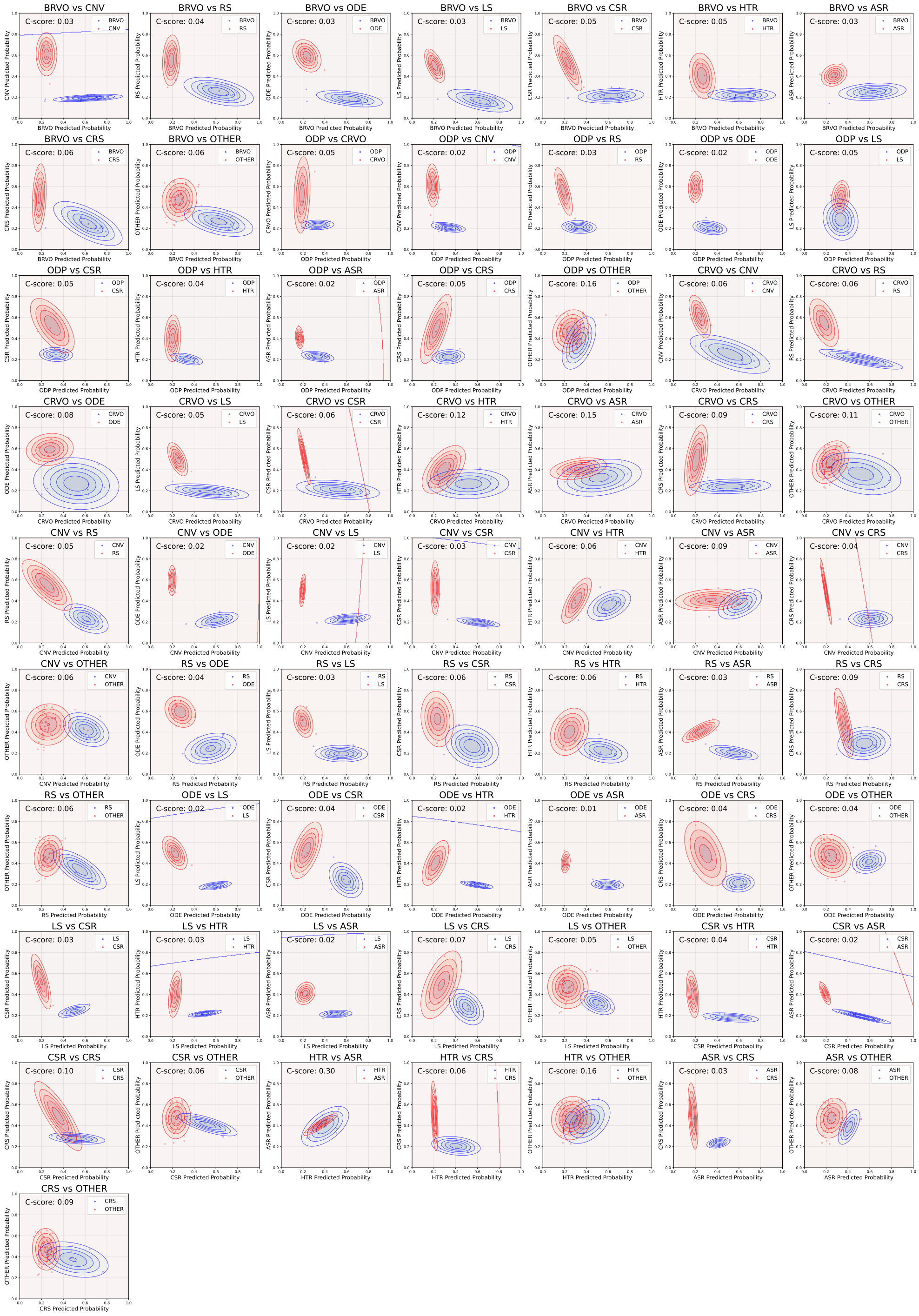

Supplementary Fig. 10: Probability scatter plots of the C-score for RetExpert's predictions on the MuReD dataset (with FDCM).(part 3/3)

Supplementary Note 1: Uncertainty-Aware Multi-Label Learning Loss Function

To enable fine-grained modeling of prediction confidence and uncertainty across individual classes, we propose a novel loss function that incorporates both Dirichlet-based evidence and class-wise uncertainty estimation. Given the model output logits $\mathbf{cls} \in \mathbb{R}^{N \times C}$, we first apply a softplus transformation to obtain non-negative evidence $\mathbf{e} \in \mathbb{R}^{N \times C}$ and define the Dirichlet parameters as $\boldsymbol{\alpha} = \mathbf{e} + 1$.

The loss function consists of two main components. The first term is the expected cross-entropy under the Dirichlet distribution, computed as:

$$\mathcal{A} = \sum_{j=1}^{C} p_j \cdot [\psi(S) - \psi(\alpha_j)], \quad (1)$$

where $S = \sum_{j=1}^{C} \alpha_j$ is the total evidence, $\psi(\cdot)$ is the digamma function, and $\mathbf{p} = [p_1, p_2, \ldots, p_C]$ denotes the one-hot or soft ground truth label, C is the number of classes.

To encourage uncertainty regularization, we introduce a KL divergence term between the predicted Dirichlet parameters and a uniform prior, scaled by an annealing coefficient λ_t:

$$\mathcal{B} = \lambda_t \cdot \mathrm{KL}(\tilde{\boldsymbol{\alpha}} \parallel \mathbf{1}), \quad \text{where } \tilde{\alpha}_j = (\alpha_j - 1)(1 - p_j) + 1, \quad (2)$$

with $\lambda_t = \min(1, t/T)$ controlling the annealing over training steps t and maximum step T. The Dirichlet-based classification loss function is then defined as:

$$\mathcal{L}_{ce} = \mathcal{A} + \mathcal{B}. \quad (3)$$

To further capture uncertainty at the class level, we define the class-wise uncertainty score as the reciprocal of the Dirichlet strength:

$$u_j = \frac{1}{\alpha_j}. \quad (4)$$

We also compute the predicted class probability using a sigmoid function: $q_j = \sigma(\alpha_j)$. Then, we formulate an uncertainty-weighted cross-entropy component:

$$\mathcal{L}_{cun} = -\sum_{j=1}^{C} u_j \cdot p_j \cdot \log(q_j + \epsilon), \quad (5)$$

where ϵ is a small constant for numerical stability. Finally, the total loss function is given by a weighted combination of the above components:

$$\mathcal{L} = \mathcal{L}_{ce} + \lambda \cdot \mathcal{L}_{cun}, \quad (6)$$

where λ is a hyperparameter to balance classification and uncertainty calibration.

This formulation enables per-class uncertainty estimation and promotes more calibrated predictions, providing a solid basis for downstream uncertainty thresholding and selective decision-making.

Algorithm 1 Uncertainty-aware multi-label learning

Require: predicted logits **cls**, ground truth label **p**, class number c, training step t, annealing step T, device

Ensure: total loss $\mathcal{L}$, per-class uncertainty score **u**

```
evidence ← Softplus(cls)                       ▷ Convert logits to non-negative evidence
α ← evidence + 1                               ▷ Construct Dirichlet parameters
S ← Σ_{j=1}^{c} α_j                            ▷ Total Dirichlet strength per sample
E ← α − 1                                      ▷ Compute adjusted evidence
A ← Σ_{j=1}^{c} p_j · [ψ(S) − ψ(α_j)]          ▷ Expected log-likelihood under Dirichlet
λ_t ← min(1, t/T)                              ▷ Compute annealing coefficient
α̃_j ← E_j · (1 − p_j) + 1                      ▷ Adjust Dirichlet for KL divergence
B ← λ_t · KL(α̃, c, device)                     ▷ KL regularization term
L_ce ← A + B                                   ▷ Dirichlet-based classification loss
u_j ← 1/α_j                                    ▷ Per-class uncertainty score
q_j ← σ(α_j)                                   ▷ Predicted class-wise probability
L_cun ← − Σ_{j=1}^{c} u_j · p_j · log(q_j + ϵ) ▷ Class-weighted uncertainty loss
λ ← 0.1                                        ▷ Weight for uncertainty penalty term
L ← mean(L_ce + λ · L_cun)                     ▷ Total uncertainty-aware loss
return total loss L, uncertainty score u
```

Supplementary Note 2: Module-Wise Progressive Test-Time Adaptation

To address the domain shift challenge inherent in deploying ophthalmic foundation models in real-world clinical scenarios, we introduce a module-wise progressive test-time adaptation method. Despite being trained on large-scale fundus datasets, models often fail to generalize to unseen data distributions due to inherited regional biases and self-supervised pretraining constraints. RetExpert performs test-time adaptation by decoupling the encoder and task-specific head, enabling targeted fine-tuning without requiring additional supervision.

This strategy consists of two sequential phases: Test-Time Unsupervised Learning (TTUL) and Test-Time Pseudo-Supervised Learning (TTPL). In TTUL, the encoder undergoes lightweight self-supervised adaptation. Specifically, only the inserted adapter modules and layer normalization layers are updated, while the task-specific head remains frozen. Given a test input x, the model prediction $p = M'(x)$ is optimized using the following loss:

$$\mathcal{L}_{\text{TTUL}} = \text{MSE}(p, x) + \text{SoftEntropy}(p) \tag{7}$$

The soft entropy regularization term is defined as:

$$\text{SoftEntropy}(\mathbf{x}) = -\sum_{i} \text{softmax}(\mathbf{x})_i \cdot \log \text{softmax}(\mathbf{x})_i \tag{8}$$

This formulation encourages confident and sharp predictions while maintaining distributional smoothness.

Following TTUL, the encoder is frozen and the model proceeds to TTPL, where the task-specific head is fine-tuned using pseudo-labels derived from the model's own predictions. From the initial prediction $p_0 = M'(x)$, we compute per-class uncertainty scores using the softplus function:

$$u_k = \frac{1}{\text{softplus}(p_{0,k}) + 1} \tag{9}$$

$$w_k = 1 - u_k \tag{10}$$

These uncertainty scores u_k are inverted into sample-wise attention weights w_k, which are then used to weight a pseudo-supervised loss. Hard pseudo-labels are obtained by thresholding:

$$\hat{y}_{0,k} = \mathbb{I}[p_{0,k} \geq \tau], \quad \text{with } \tau = 0.5 \tag{11}$$

where $\mathbb{I}$ is indicator function. The loss used for TTPL is defined as:

$$\mathcal{L}_{\text{TTPL}} = \text{WBCE}(\hat{y}_0, p_0; w) + \text{SoftEntropy}(p_0) \tag{12}$$

Here, WBCE denotes the weighted binary cross-entropy, defined as:

$$\mathrm{WBCE}(\hat{y}_0, p_0; w) = \frac{1}{N}\sum_{n=1}^{N}\frac{1}{C}\sum_{c=1}^{C} w_{n,c}\left[-\hat{y}_{0,n,c}\cdot\log\sigma(p_{0,n,c}) - (1-\hat{y}_{0,n,c})\cdot\log(1-\sigma(p_{0,n,c}))\right] \tag{13}$$

where $\sigma(\cdot)$ is the sigmoid function, $w_{n,c}$ is the class-wise weight for sample n, C is the number of classes, and N is the batch size. The TTPL step is executed over I iterations using SGD with a learning rate of 1×10^{-3} and momentum of 0.9.

In summary, this dual-phase adaptation strategy enables the model to exploit domain-specific information at test time, enhancing generalization without supervision. This process is efficient and deployable on a per-sample basis.

Algorithm 2 Module-wise progressive test-time adaptation (TTUL + TTPL)

Require: Pretrained model M, test sample x, adaptation mode finetune_mode, iteration steps K (unsupervised) and I (pseudo-supervised)
Ensure: Adapted prediction $\hat{y}$

Initialize: Clone model M' from M
Load parameters from M to M'
Set M' to evaluation mode
▷ *Configure trainable parameters according to finetune mode*
for all parameter (θ, name) in M' **do**
    if name matches finetune_mode **then**
        θ.requires_grad $\leftarrow$ True
    else
        θ.requires_grad $\leftarrow$ False
    end if
end for
Define optimizer $\mathcal{O}_1$ over selected parameters
Define optimizer $\mathcal{O}_2$ for head parameters
▷ **Phase I: Test-Time Unsupervised Learning (TTUL)**
for $i = 1 \to K$ **do**
    Predict output $p = M'(x)$
    Compute unsupervised loss $\mathcal{L}_{\mathrm{TTUL}} = \mathcal{L}_{\mathrm{MAE}} + \mathrm{SoftEntropy}(p)$
    Backpropagate $\mathcal{L}_{\mathrm{TTUL}}$ and update parameters with $\mathcal{O}_1$
end for
▷ **Phase II: Test-Time Pseudo-Supervised Learning (TTPL)**
Freeze encoder; unfreeze task-specific head
Predict output $p_0 = M'(x)$
Compute uncertainty scores $u = 1/(\texttt{Softplus}(p_0) + 1)$
Derive confidence weights $w = 1 - u$
Generate pseudo-label $\hat{y}$ by thresholding τ
for $j = 1 \to I$ **do**
    Predict output $p_0 = M'(x)$
    Compute loss:

$$\mathcal{L}_{\mathrm{TTPL}} = \mathrm{WBCE}(\hat{y}, p_0; w) + \mathrm{SoftmaxEntropy}(p_0))$$

    Backpropagate $\mathcal{L}_{\mathrm{TTPL}}$ and update parameters with $\mathcal{O}_2$
end for
▷ **Final Inference**
Predict $\hat{y} = M'(x)$
return Adapted prediction $\hat{y}$

Supplementary Note 3: Inference Time and Computational Cost

The computational cost assessment for our proposed test-time adaptation method, including inference time, throughput, and GPU usage, is presented in **Supplementary Table 1**. Specifically, GPU memory usage for all methods (Baseline, TTUL, TTPL, and TTUL+TTPL) ranges from 4.23 GB to 4.61 GB and remains at comparable levels during the inference phase. In terms of inference time, the proposed test-time adaptation method (TTUL+TTPL) incurs an additional 0.13 seconds compared to the Baseline configuration. The TTUL+TTPL method still maintains a detection efficiency above 3 FPS. Despite a slightly longer inference time, our combined TTUL+TTPL method improves the Kappa value by over 9% in both test scenarios: MuReD-to-ODIR and ODIR-to-MuReD).

Supplementary Table 1: Inference efficiency comparison of different methods.

**Method**	**Inference Time (s)** ↓	**Throughput (FPS)** ↑	**GPU Usage (GB)** ↓
Baseline	0.1744	5.7318	4.2324
TTUL	0.3018	3.3134	4.3945
TTPL	0.2338	4.2766	4.2402
TTUL+TTPL	0.3051	3.2767	4.3945

Supplementary Note 4: Supplementary Description of Datasets for Validation

We discussed the potential overlap or duplication in the fundus datasets utilized in the study.

Regarding the 9 public datasets used in our study, we have meticulously examined their data sources, population information, and disease cohorts. Among them, DRarranged, Drishti-GS, HRF, and OTFID originated from different countries, without any overlap or patient-level duplication. As for the publicly available datasets from China, MMAC was sourced from the Shanghai Health and Medical Center and Shanghai Sixth People's Hospital in China, while iChallengeGON was collected from several hospitals and clinical studies. Since the two datasets are derived from separate regions within China, it can be concluded that there is no patient-level duplication. The ADAM, GAMMA, and PALM datasets all originated from the Zhongshan Ophthalmic Center, Sun Yat-sen University, China. While we cannot definitively confirm that there is no patient-level duplication among these datasets, they can be considered "unknown" in that regard. However, they were derived from distinct disease cohorts, making them suitable for validating the performance of our multi-disease detection model. As for the 6 private datasets, STDR, Vietnam, AITS, CUHK-GON(D/M), and Hand-Held, they were collected from different regions or studies, and there is no overlap or patient-level duplication across any of these datasets. However, CUHK-GON(D) and CUHK-GON(M) were derived from the same study and involved patient-level duplication. The distinction between these two datasets lies in the difference in the field of view (FOV) of the images, but there is no image-level duplication.

For the proposed RetExpert, the test datasets presented in **Supplementary Table 2** and **Supplementary Table 3** constitute out-of-distribution (OOD) fundus images originating from genuinely unseen populations and imaging protocols. The proposed RetExpert was trained on datasets (e.g., MuReD), which comprises populations from USA, UK, and India, and images captured by five device types: TOPCON TRV-50, TOPCON TRC-NW300, ZEISS FF450, TOPCON 3D OCT-2000, and Kowa VX-10α. Therefore, for validation on retrospective unseen datasets, we employed two types of OOD data to evaluate the real-world robustness of RetExpert. The first type is population OOD datasets, which include public datasets (ADAM, iChallenge-GON, PALM, GAMMA, and MMAC) from Chinese populations, as well as private datasets from China and Vietnam. The second type is device OOD datasets, which include the public datasets HRF (Canon CF-60UVi) and OTFID (VISUCAM 500; Pictor Plus-Portable Ophthalmic Camera), as well as the private dataset Hand-Held (ZhenHealth, PANDA). This study has demonstrated that our proposed RetExpert outperformed not only conventional models but also related foundation models (FMs) in multi-disease detection, with particularly marked gains on various unseen OOD datasets.

Supplementary Table 2: Description of publicly available CFP datasets for Validation.

Dataset	Disease	Volume	Camera Type	Region	Age	FOV	Resolution	Patient-level Overlap
DRarranged	N;DR	35127	Centervue DRS; Optovue iCam; Canon CR1/DGi/CR2; Topcon NW	USA; India	54.40(11.30)	Disc/macular center	Hybrid	No
ADAM	N;AMD	400	Zeiss Visucam 500	China	53.19(15.59)	Macular center	2124×2056; 1444×1444	Unknown
iChallengeGON	N;GL	400	Zeiss Visucam 500; Canon CR-2	China	25.30(11.50)	Macular center	2124×2056	No
PALM	N;MYA	374	Zeiss Visucam 500; Canon CR-2	China	37.50(15.91)	Disc/macular center	2124×2056; 1444×1444	Unknown
Drishti-GS	N;GL	101	–	India	40–80	Disc center	2896×1944	No
HRF	N;DR;GL	45	Canon CF-60UVi	Czech Republic	–	Disc/macular center	3504×2336	No
OTFID	N;OT	411	VISUCAM 500; Pictor Plus-Portable Ophthalmic Camera	Paraguay	<18	Disc/macular center	2124×2056; 1536×1152	No
GAMMA	N;GL	100	KOWA; Topcon TRC-NW400	China	19–77	Macular center	2000×2992; 1934×1956	Unknown
MMAC	N;MYA	1143	Topcon TRC-NW400	China	52.22(9.16); 64.40(12.38)	Macular center	800×800	No

N: normal; DR: diabetic retinopathy; AMD: age-related macula degeneration; GL: glaucoma; MYO: myopia; OT: ocular toxoplasmosis.

Supplementary Table 3: Description of private CFP datasets for validation.

Dataset	Disease	Volume	Camera Type	Region	Age	FOV	Resolution	Patient-level Overlap
STDR	N;DR	11157	Topcon TRC-50DX	China	67.73(13.54)	Disc/macular center	3696×2448	No
Vietnam	N;DR	3880	Carl Zeiss Cirrus	Vietnam	62.67(9.87)	Disc/macular center	2976×1984; 512×512	No
AITS	N;DR	1053	Topcon NW500; Topcon Triton	China	64.65(9.37)	Disc/macular center	2676×2676; 1960×1934	No
CUHK-GON(D)	N;GL	5063	Topcon TRC-50DX	China	59.58(10.12)	Disc center	3696×2448	Yes
CUHK-GON(M)	N;GL	1701	Topcon TRC-50DX	China	59.58(10.12)	Macular center	3696×2448	Yes
Hand-Held	N;DR; TSLN;DN MYA;O	553	ZhenHealth PANDA	China	41.99(12.75)	Macular center	1531×1531	No

N: normal; DR: diabetic retinopathy; GL: glaucoma; TSLN: tessellation; DN: drusens; MYO: myopia; O: others.
